# Supplementary material for: Reprogramming of cardiac phosphoproteome, proteome, and transcriptome confers resilience to chronic adenylyl cyclase-driven stress
Source: eLife. 2024 Jan 22;12:RP88732. doi: 10.7554/eLife.88732 (PMC10945681; doi:10.7554/eLife.88732)
Supplement: Figure 6—source data 1. [file elife-88732-fig6-data1.zip › AKT Phosphorylation.pptx]

## Slide 1
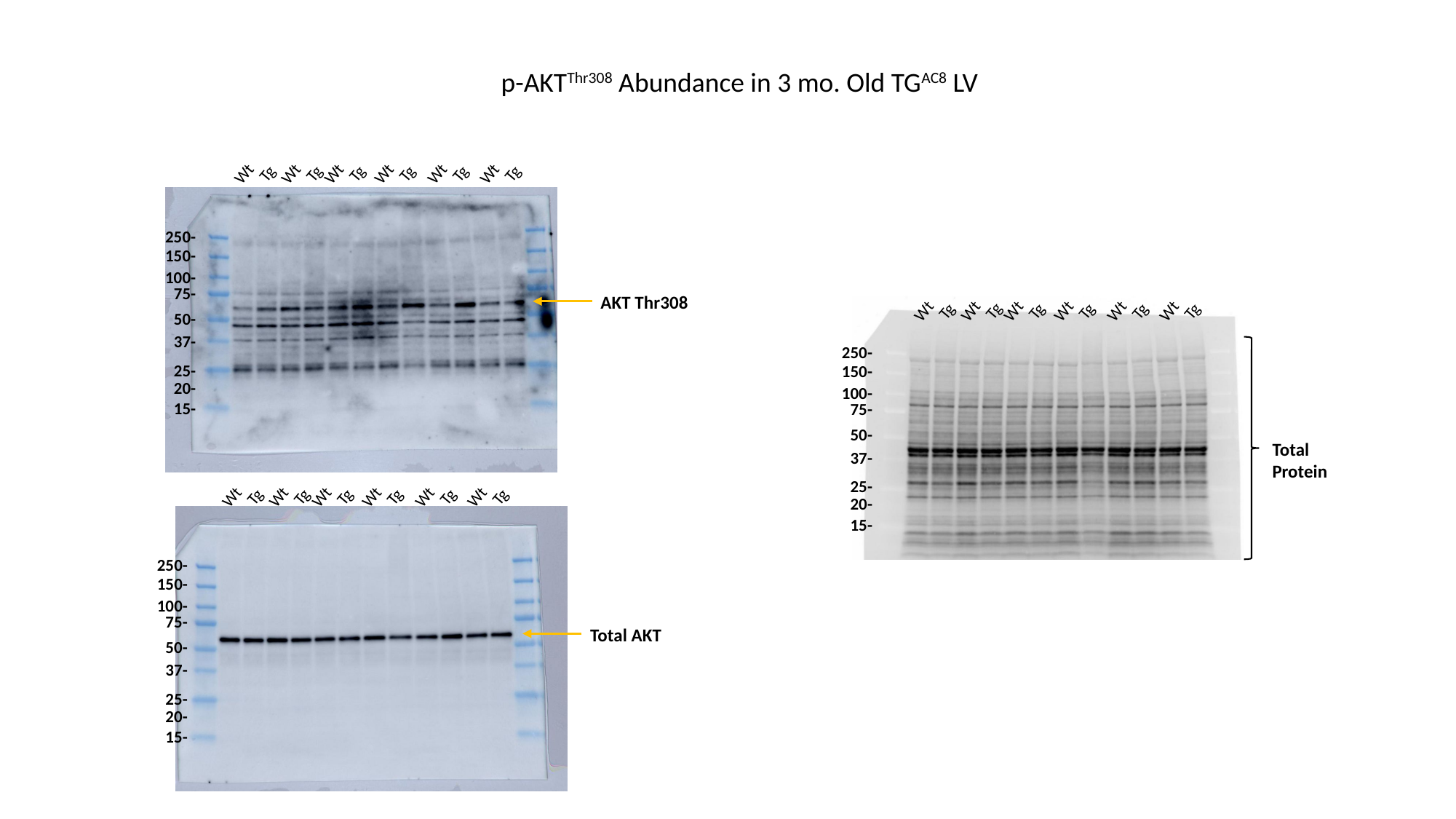

p-AKTThr308 Abundance in 3 mo. Old TGAC8 LV
Wt
Tg
Wt
Tg
Wt
Tg
Wt
Tg
Wt
Tg
Wt
Tg
250-
150-
100-
75-
50-
37-
25-
20-
15-
AKT Thr308
Wt
Tg
Wt
Tg
Wt
Tg
Wt
Tg
Wt
Tg
Wt
Tg
250-
150-
100-
75-
50-
37-
25-
20-
15-
Total Protein
Wt
Tg
Wt
Tg
Wt
Tg
Wt
Tg
Wt
Tg
Wt
Tg
250-
150-
100-
75-
50-
37-
25-
20-
15-
Total AKT

## Slide 2
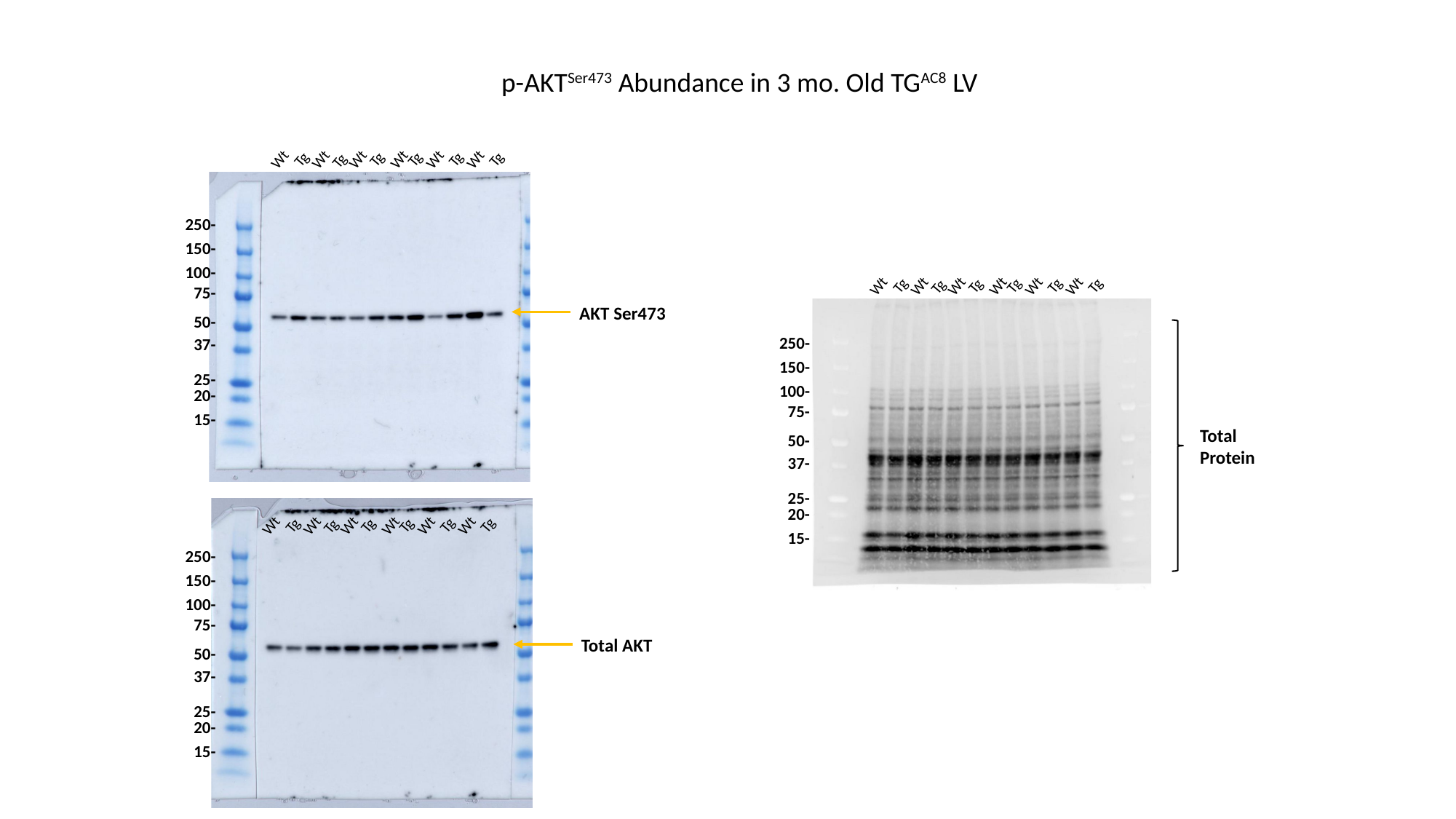

p-AKTSer473 Abundance in 3 mo. Old TGAC8 LV
Wt
Tg
Wt
Wt
Tg
Wt
Tg
Wt
Tg
Wt
Tg
Tg
250-
150-
100-
75-
50-
37-
25-
20-
15-
Wt
Tg
Wt
Wt
Tg
Wt
Tg
Wt
Tg
Wt
Tg
Tg
AKT Ser473
250-
150-
100-
75-
50-
37-
25-
20-
15-
Total Protein
Wt
Tg
Wt
Wt
Tg
Wt
Tg
Wt
Tg
Wt
Tg
Tg
250-
150-
100-
75-
50-
37-
25-
20-
15-
Total AKT
