# Supplementary figures and images for: Reprogramming of cardiac phosphoproteome, proteome, and transcriptome confers resilience to chronic adenylyl cyclase-driven stress
Source: eLife. 2024 Jan 22;12:RP88732. doi: 10.7554/eLife.88732 (PMC10945681; doi:10.7554/eLife.88732)

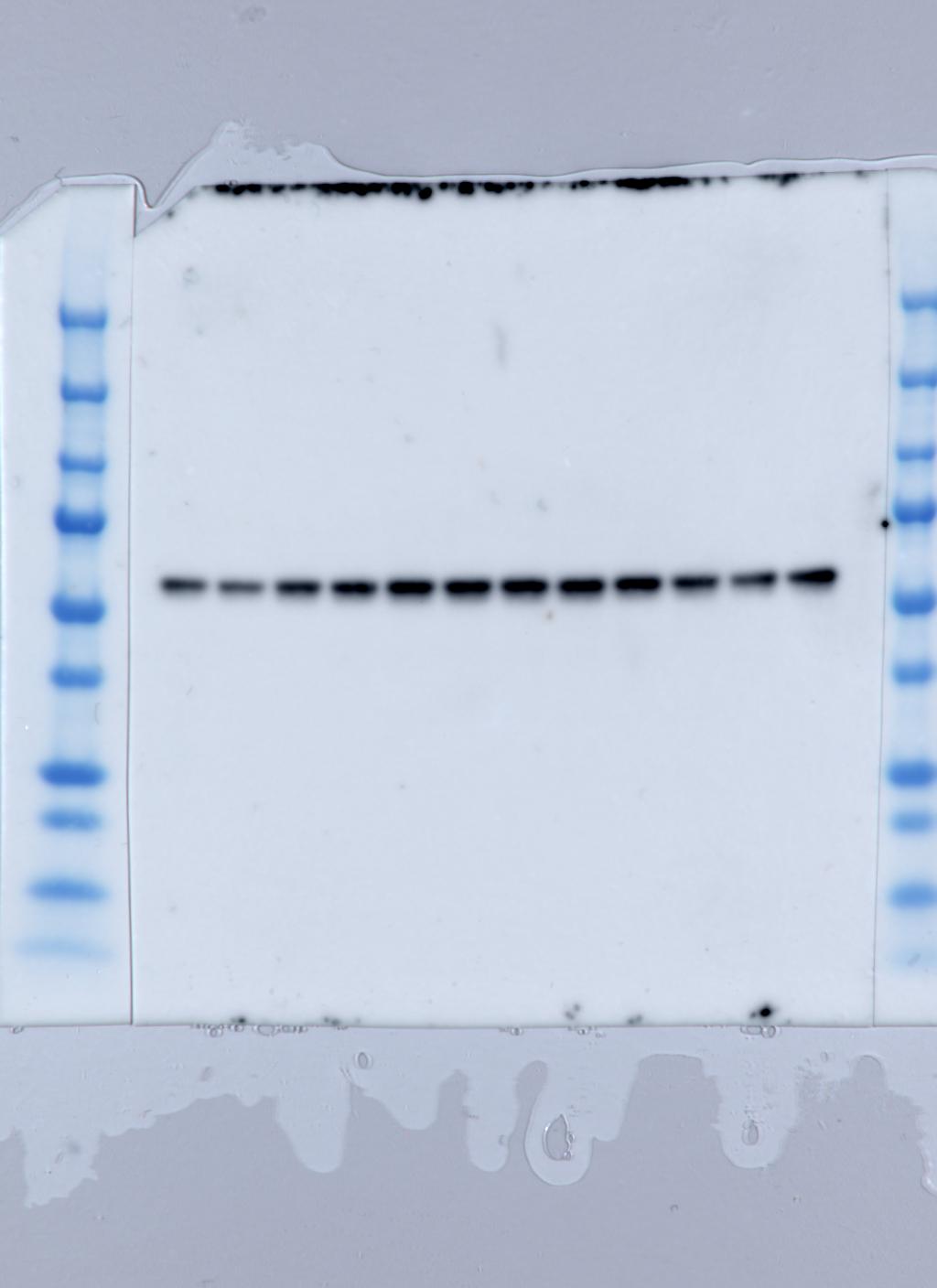

Supplement: Figure 6—source data 1. [file elife-88732-fig6-data1.zip › p-AKT Ser473/DR AKT Blot62 2020.03.04_10.52.21_Ch/DR AKT Blot62 2020.03.04_10.52.21_Ch+Marker.jpg]

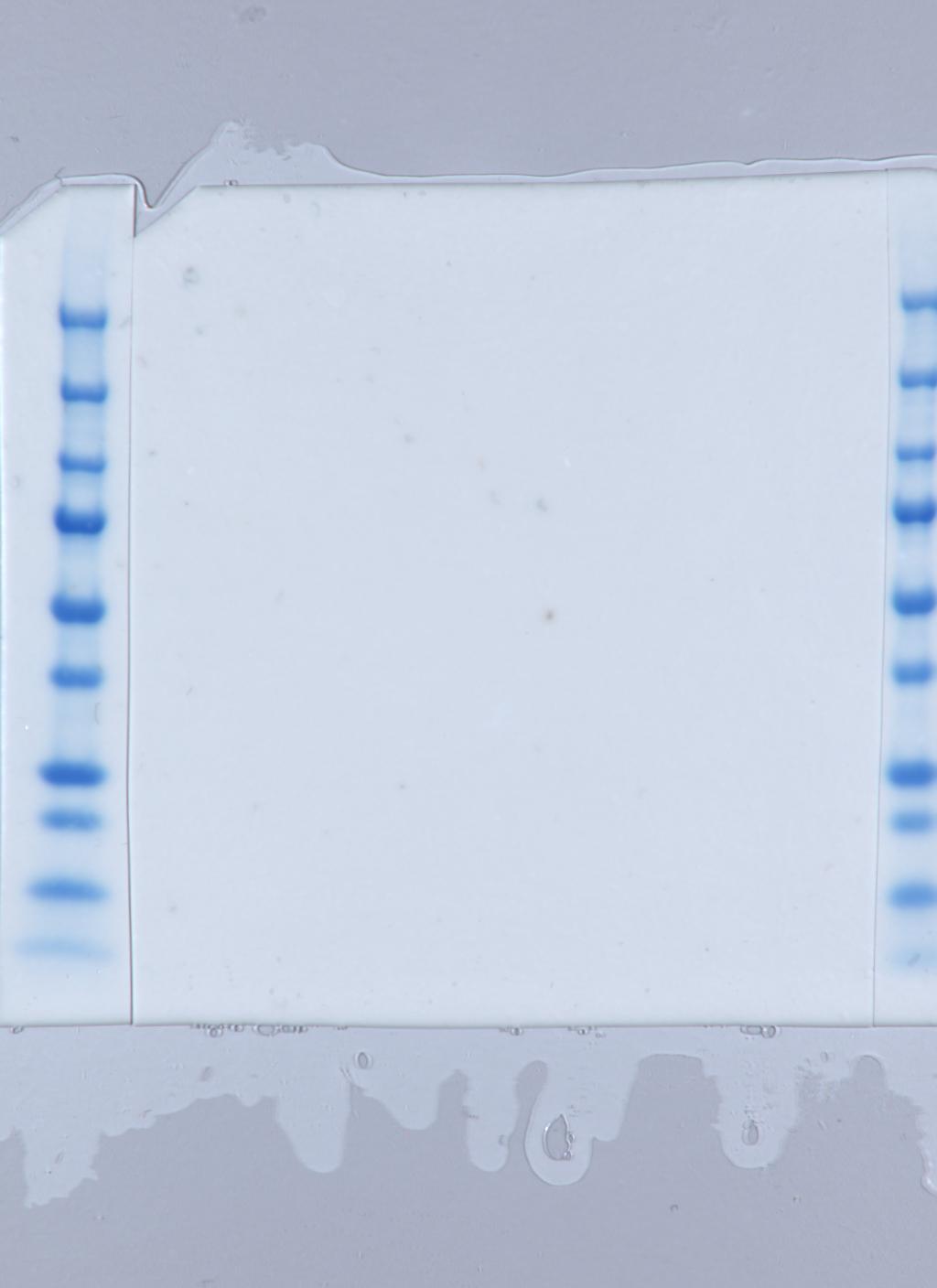

Supplement: Figure 6—source data 1. [file elife-88732-fig6-data1.zip › p-AKT Ser473/DR AKT Blot62 2020.03.04_10.52.21_Ch/DR AKT Blot62 2020.03.04_10.52.21_Ch-Marker.jpg]

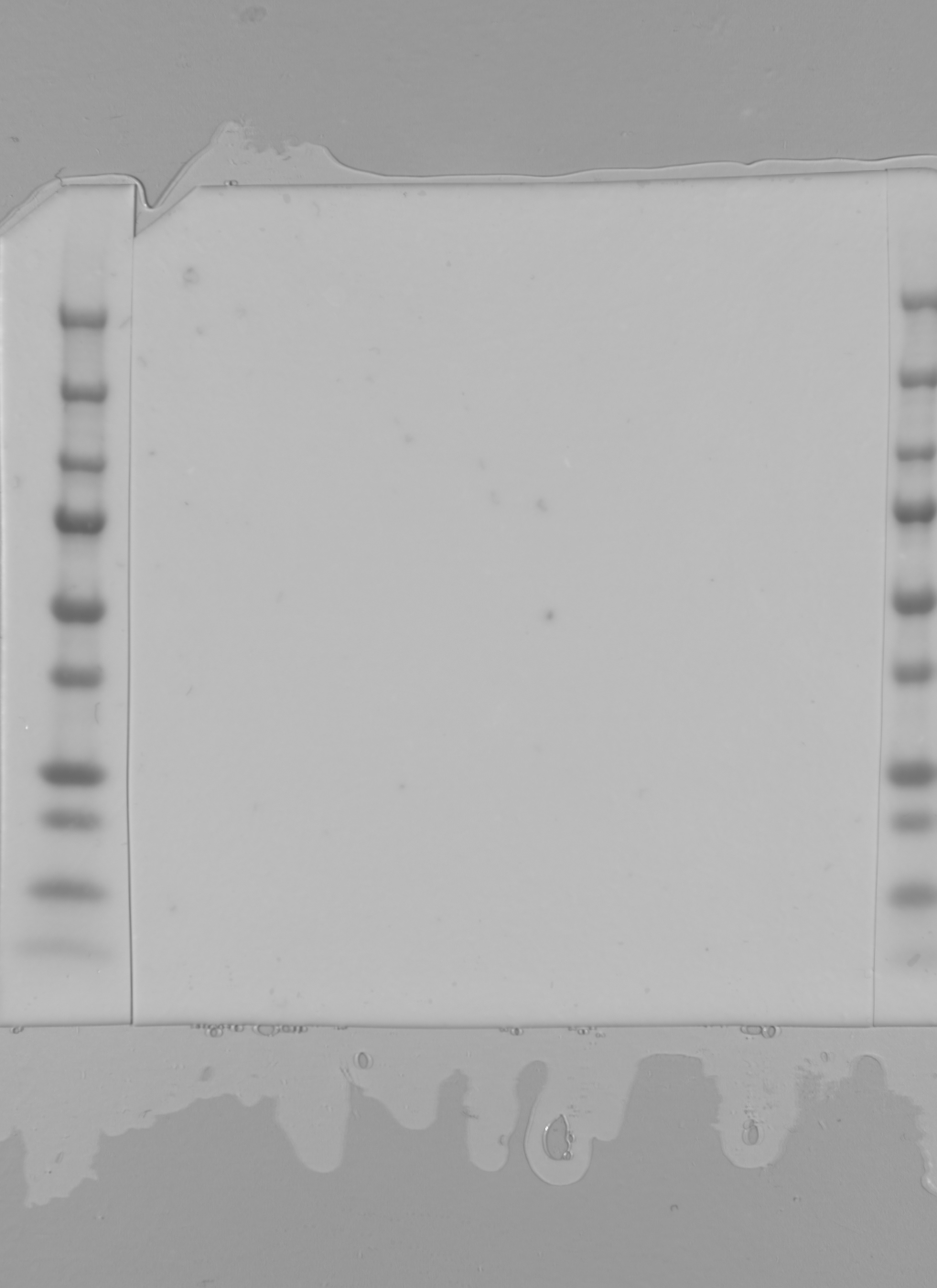

Supplement: Figure 6—source data 1. [file elife-88732-fig6-data1.zip › p-AKT Ser473/DR AKT Blot62 2020.03.04_10.52.21_Ch/DR AKT Blot62 2020.03.04_10.52.21_Ch-Marker.tif]

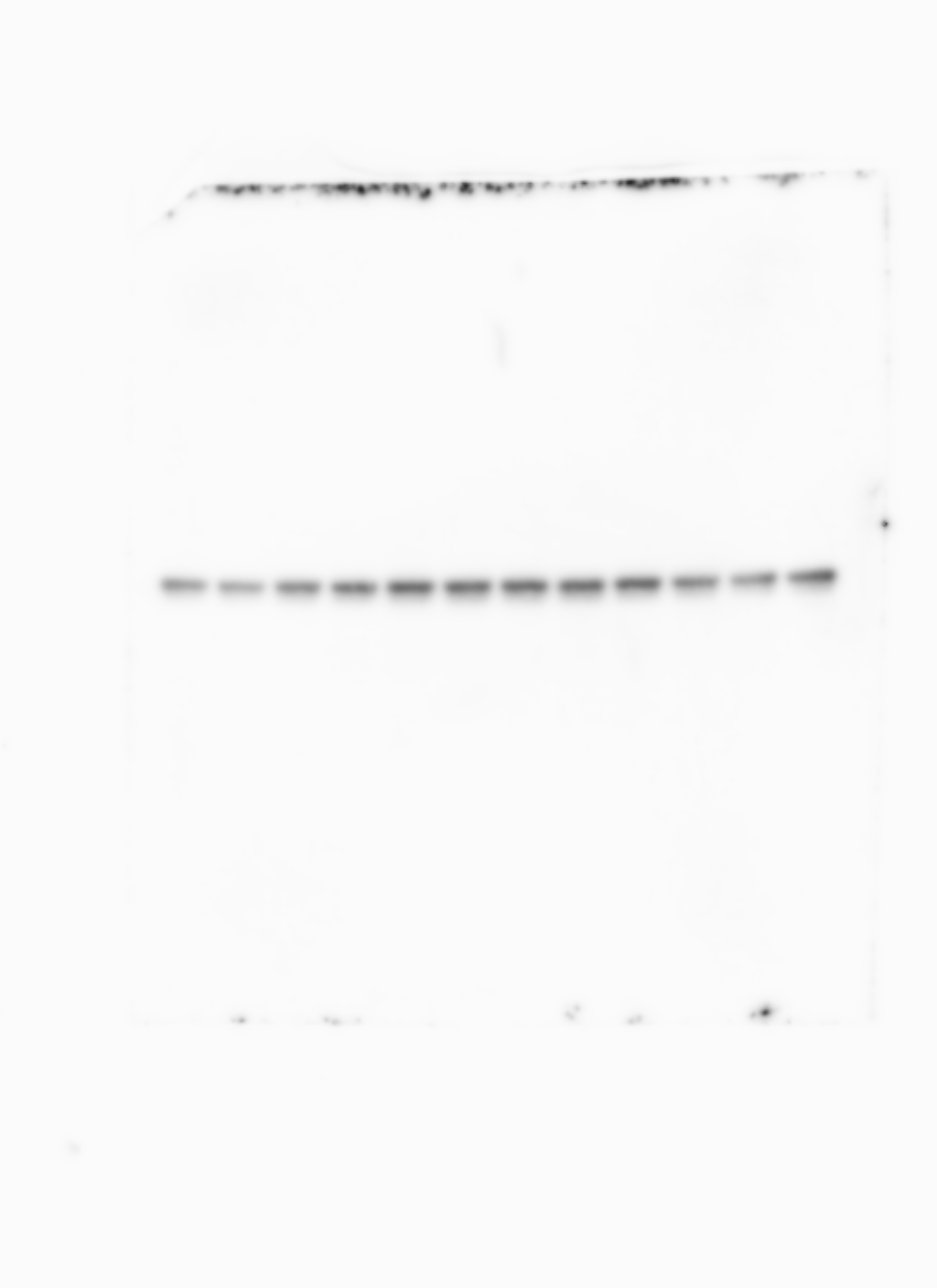

Supplement: Figure 6—source data 1. [file elife-88732-fig6-data1.zip › p-AKT Ser473/DR AKT Blot62 2020.03.04_10.52.21_Ch/DR AKT Blot62 2020.03.04_10.52.21_Ch.tif]

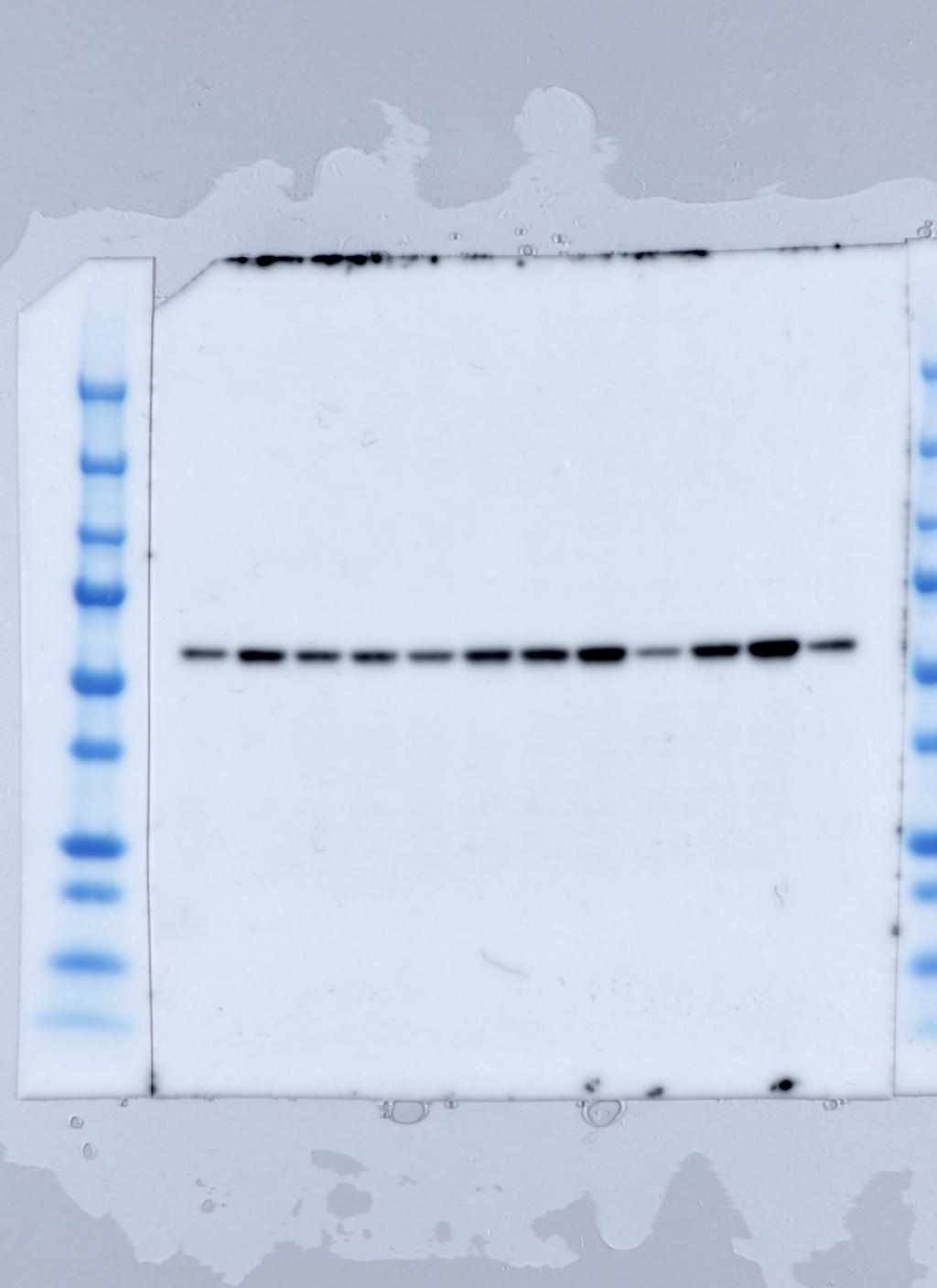

Supplement: Figure 6—source data 1. [file elife-88732-fig6-data1.zip › p-AKT Ser473/DR pAKT S473 Blot62 2020.03.03_12.28.29_Ch/DR pAKT S473 Blot62 2020.03.03_12.28.29_Ch+Marker.jpg]

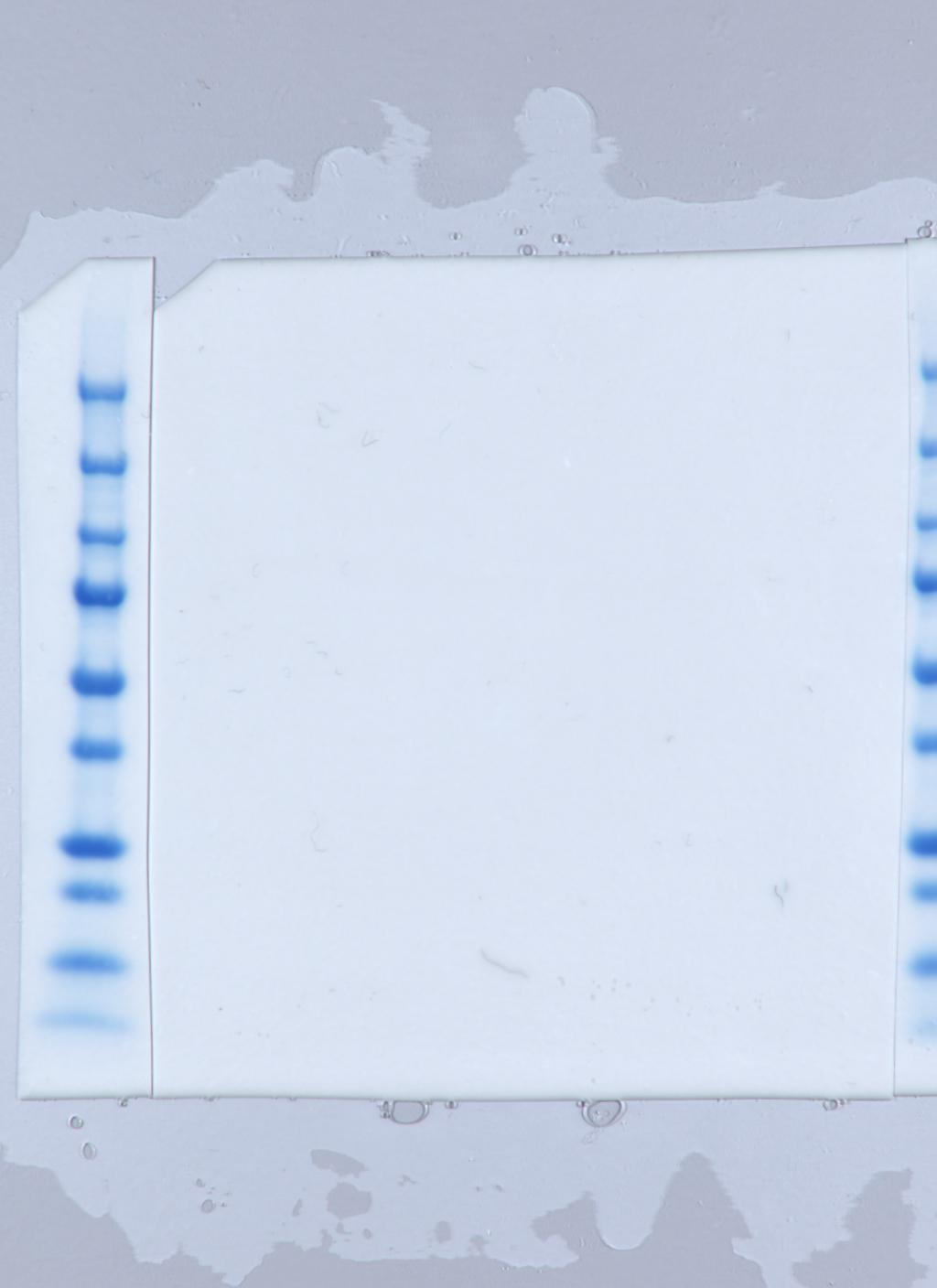

Supplement: Figure 6—source data 1. [file elife-88732-fig6-data1.zip › p-AKT Ser473/DR pAKT S473 Blot62 2020.03.03_12.28.29_Ch/DR pAKT S473 Blot62 2020.03.03_12.28.29_Ch-Marker.jpg]

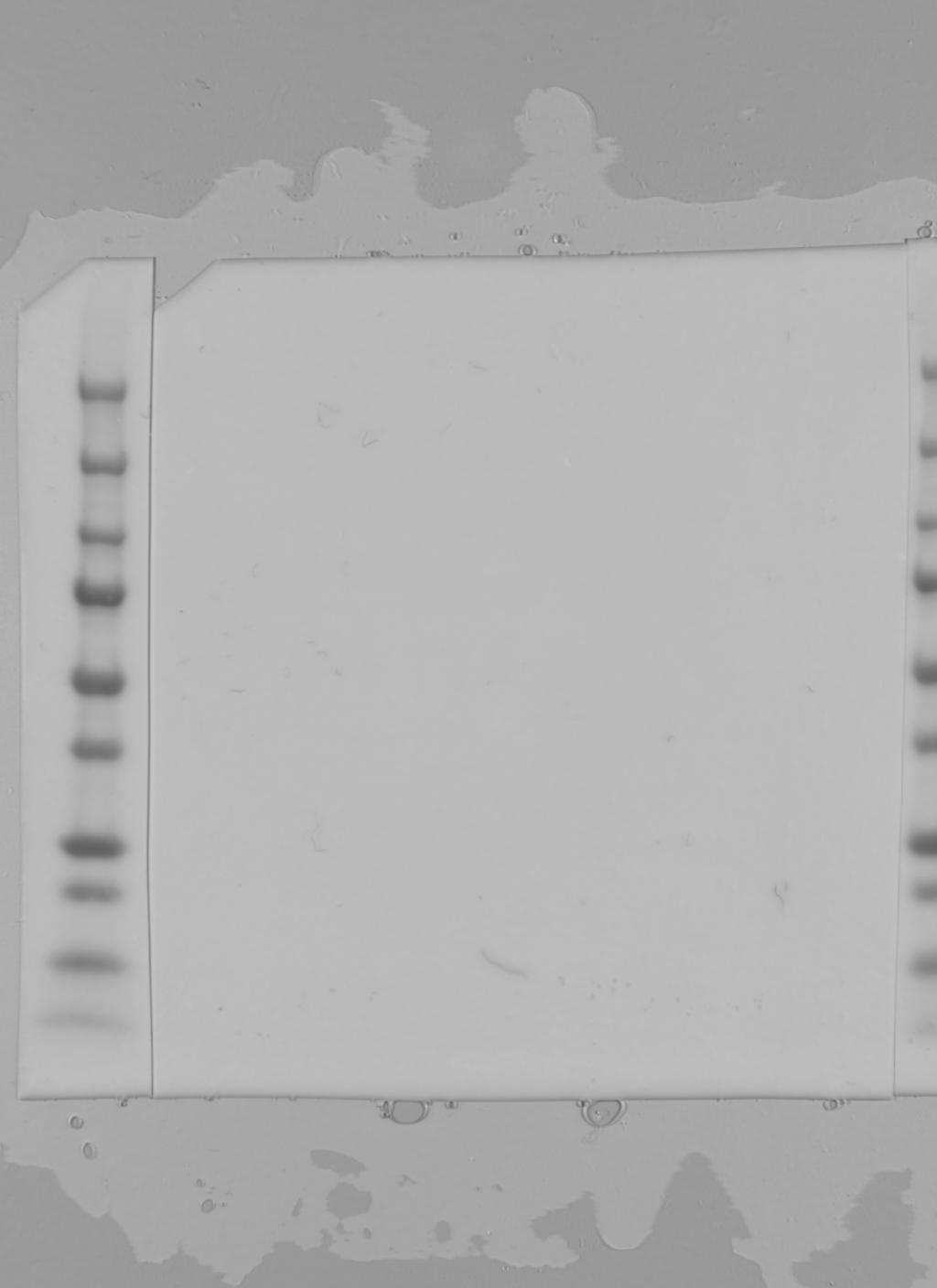

Supplement: Figure 6—source data 1. [file elife-88732-fig6-data1.zip › p-AKT Ser473/DR pAKT S473 Blot62 2020.03.03_12.28.29_Ch/DR pAKT S473 Blot62 2020.03.03_12.28.29_Ch-Marker.tif]

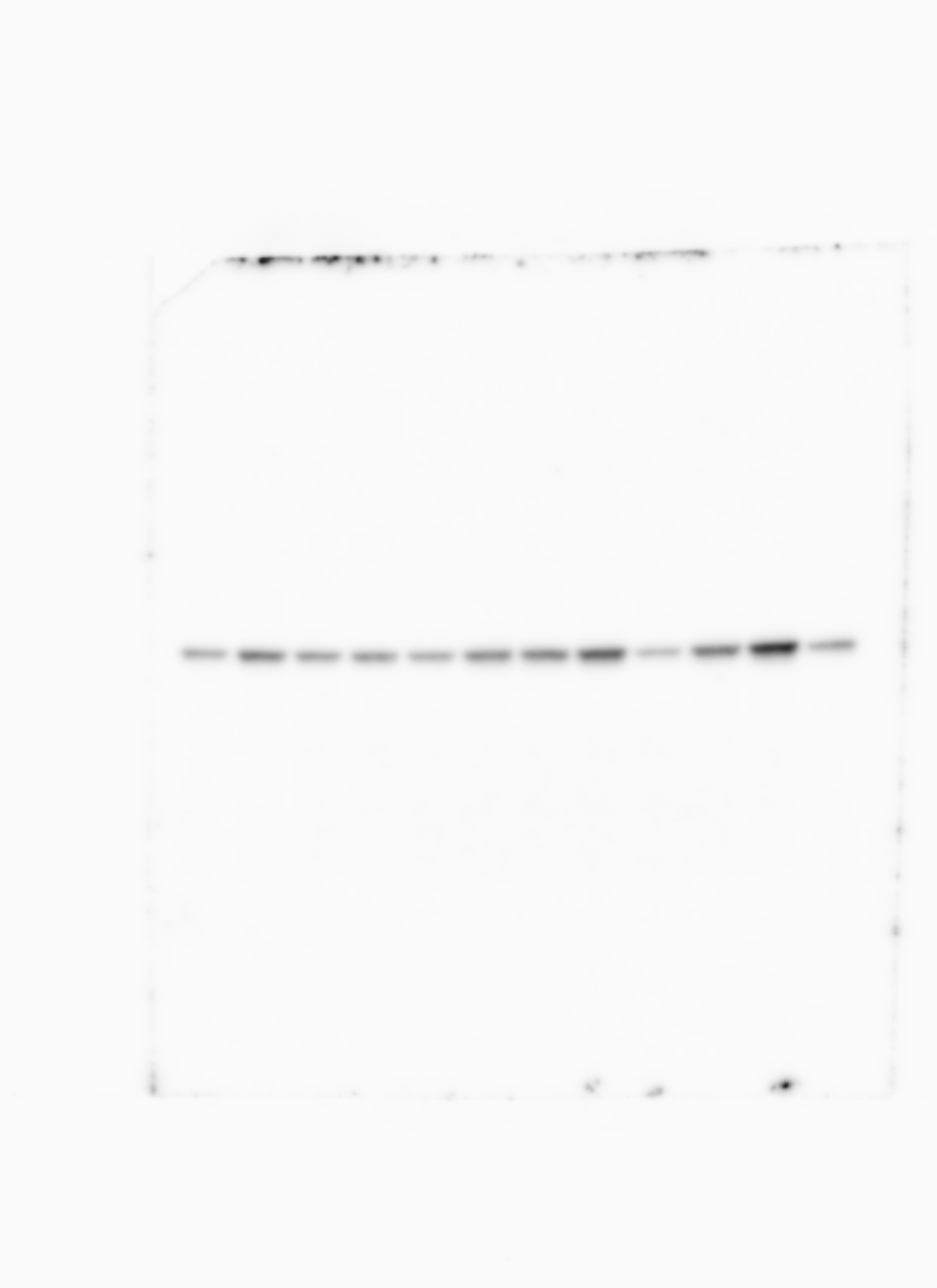

Supplement: Figure 6—source data 1. [file elife-88732-fig6-data1.zip › p-AKT Ser473/DR pAKT S473 Blot62 2020.03.03_12.28.29_Ch/DR pAKT S473 Blot62 2020.03.03_12.28.29_Ch.tif]

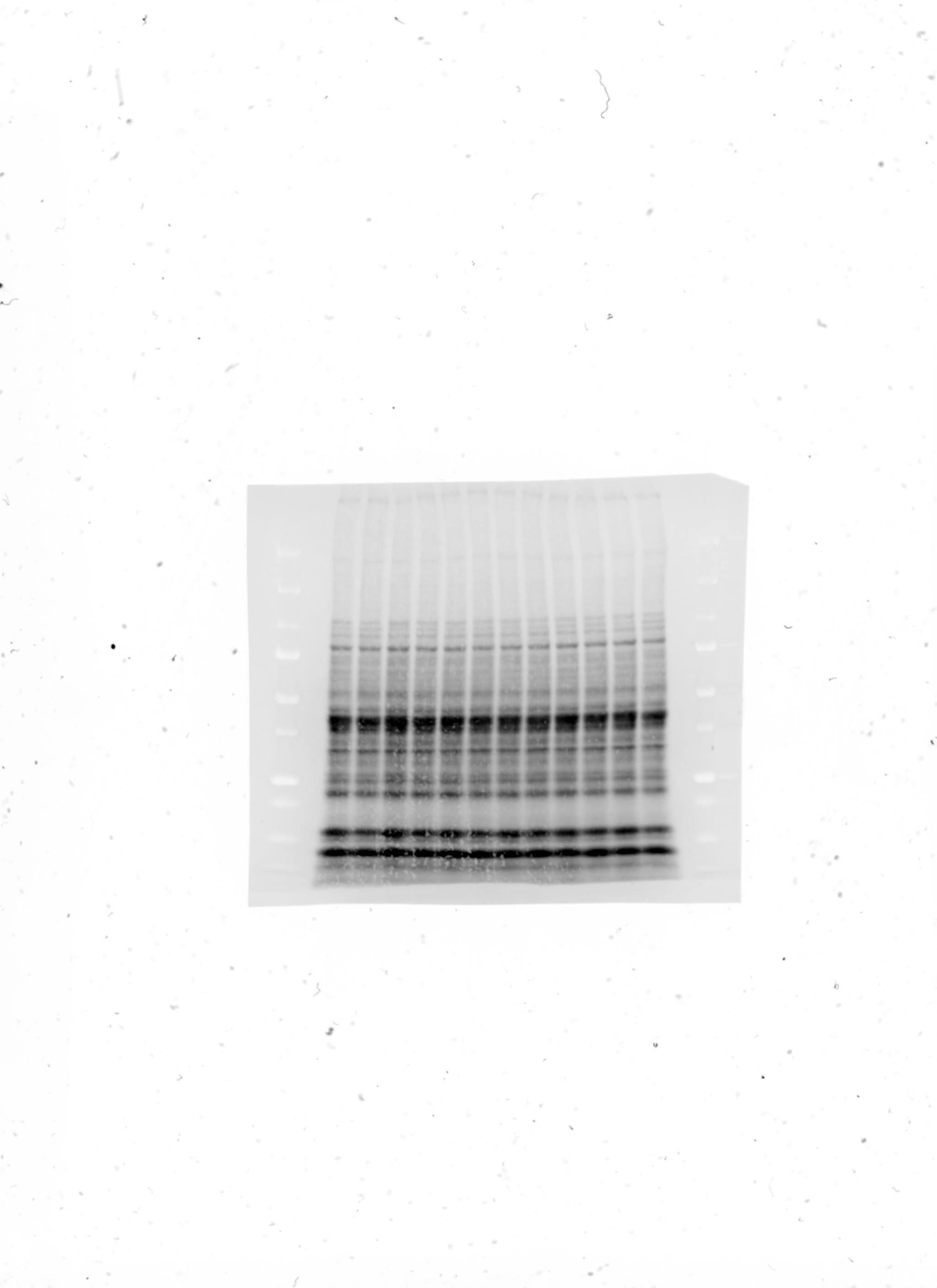

Supplement: Figure 6—source data 1. [file elife-88732-fig6-data1.zip › p-AKT Ser473/DR T.Prot Blot62 2020.03.02_13.16.56_Fl-UV/DR T.Prot Blot62 2020.03.02_13.16.56_Fl-UV.jpg]

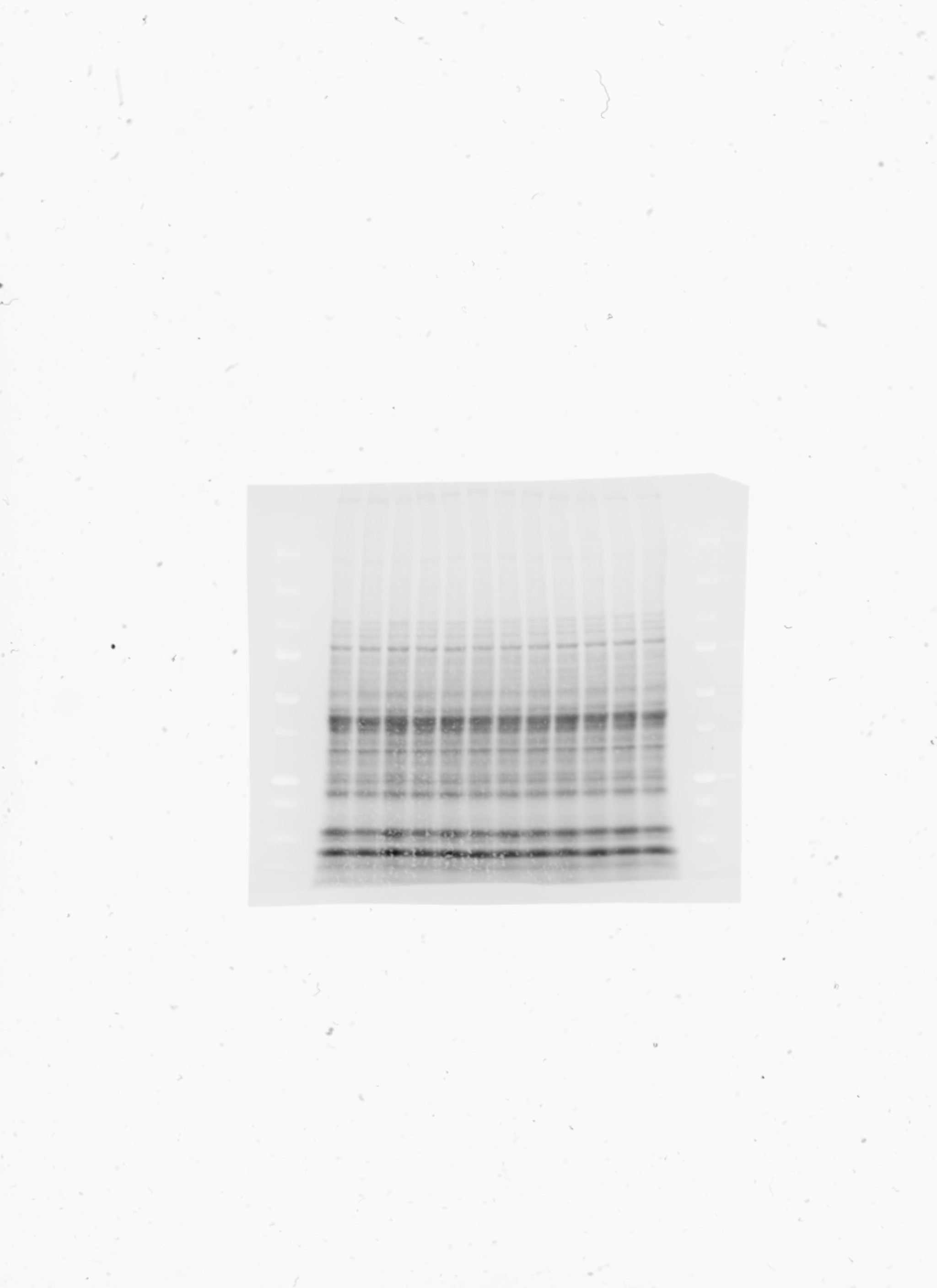

Supplement: Figure 6—source data 1. [file elife-88732-fig6-data1.zip › p-AKT Ser473/DR T.Prot Blot62 2020.03.02_13.16.56_Fl-UV/DR T.Prot Blot62 2020.03.02_13.16.56_Fl-UV.tif]

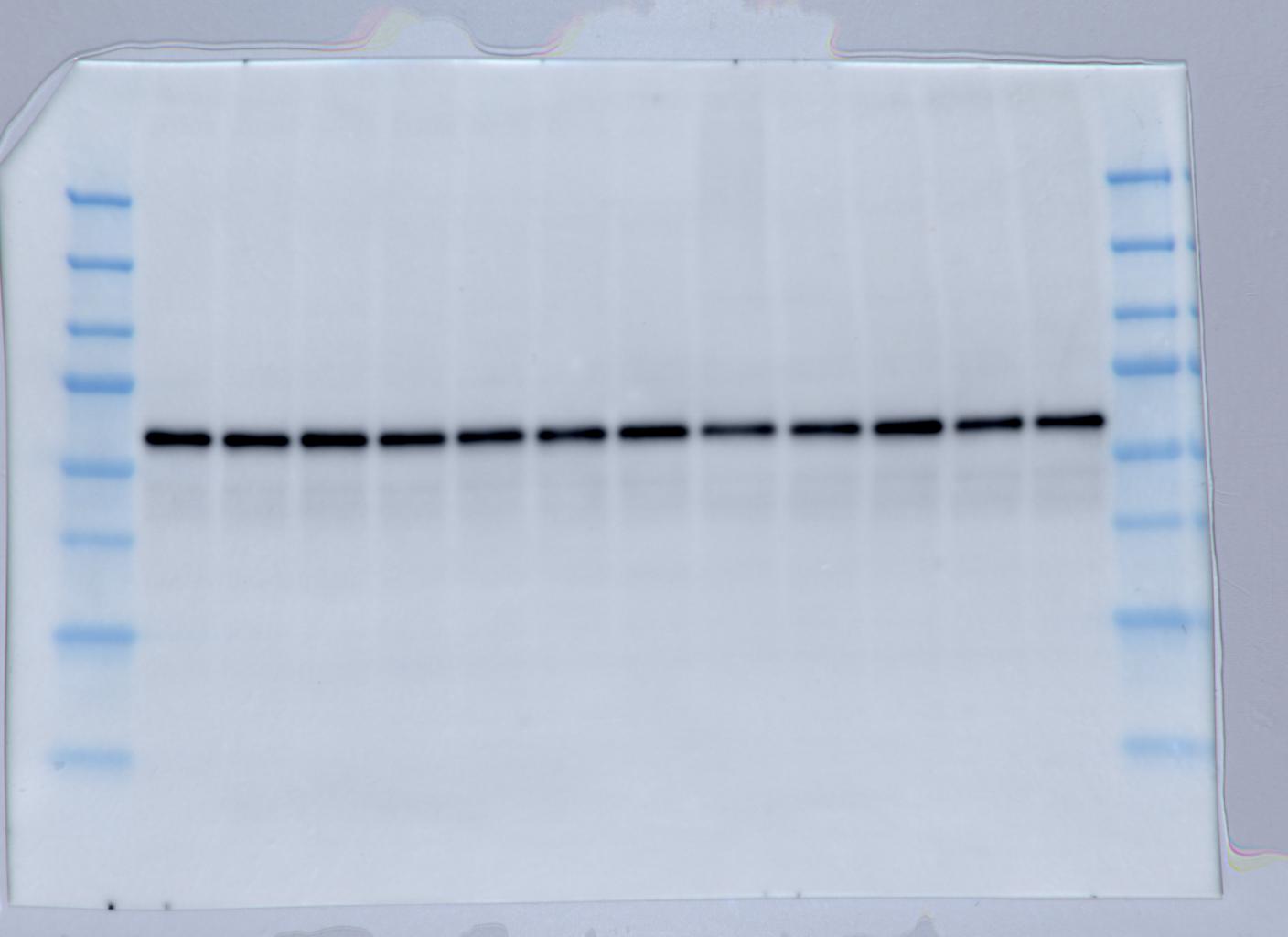

Supplement: Figure 6—source data 1. [file elife-88732-fig6-data1.zip › p-AKT Thr308/DR Akt Blot3 WPP 2018.02.01_12.07.20_Ch/DR Akt Blot3 WPP 2018.02.01_12.07.20_Ch+Marker.jpg]

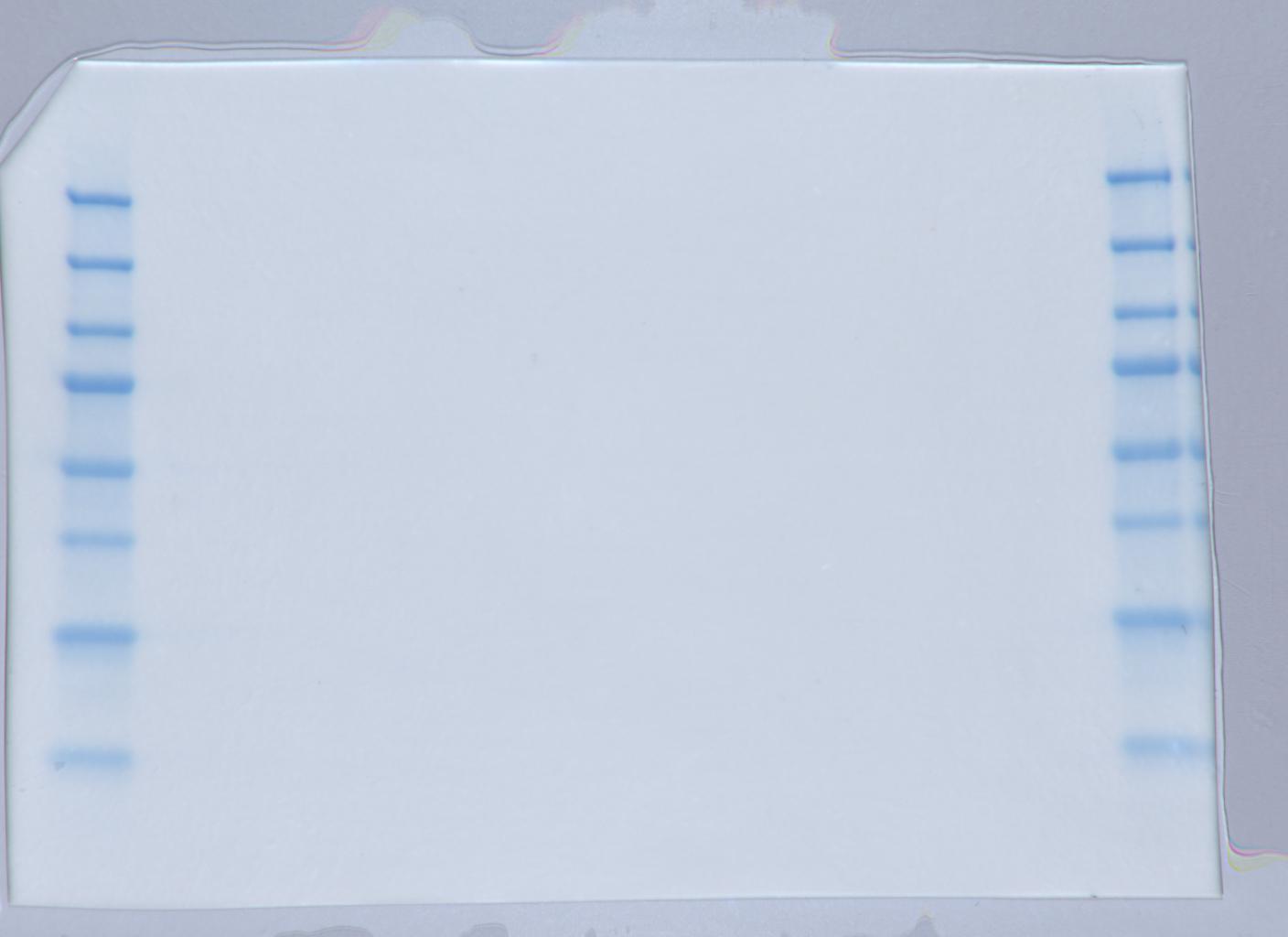

Supplement: Figure 6—source data 1. [file elife-88732-fig6-data1.zip › p-AKT Thr308/DR Akt Blot3 WPP 2018.02.01_12.07.20_Ch/DR Akt Blot3 WPP 2018.02.01_12.07.20_Ch-Marker.jpg]

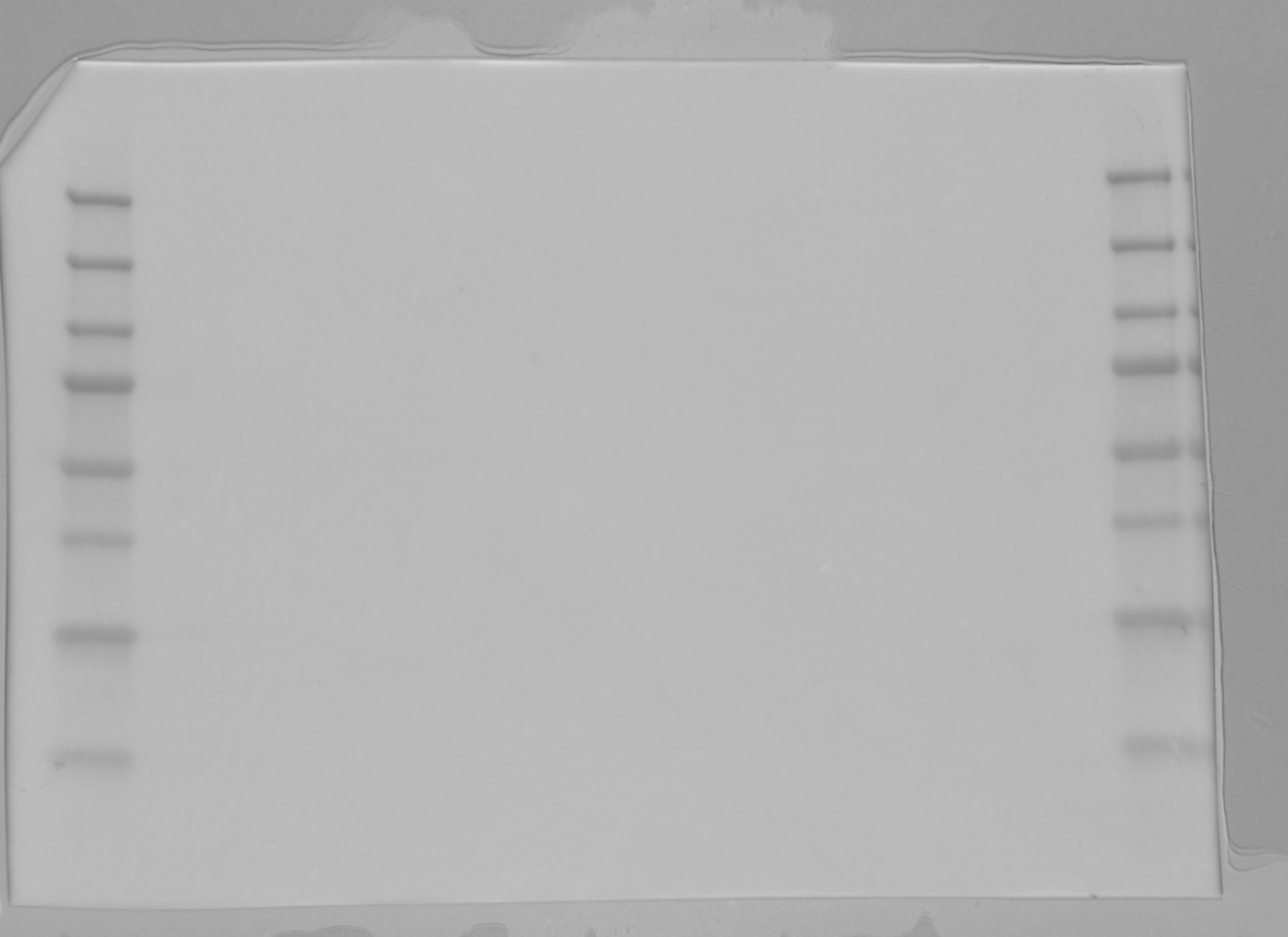

Supplement: Figure 6—source data 1. [file elife-88732-fig6-data1.zip › p-AKT Thr308/DR Akt Blot3 WPP 2018.02.01_12.07.20_Ch/DR Akt Blot3 WPP 2018.02.01_12.07.20_Ch-Marker.tif]

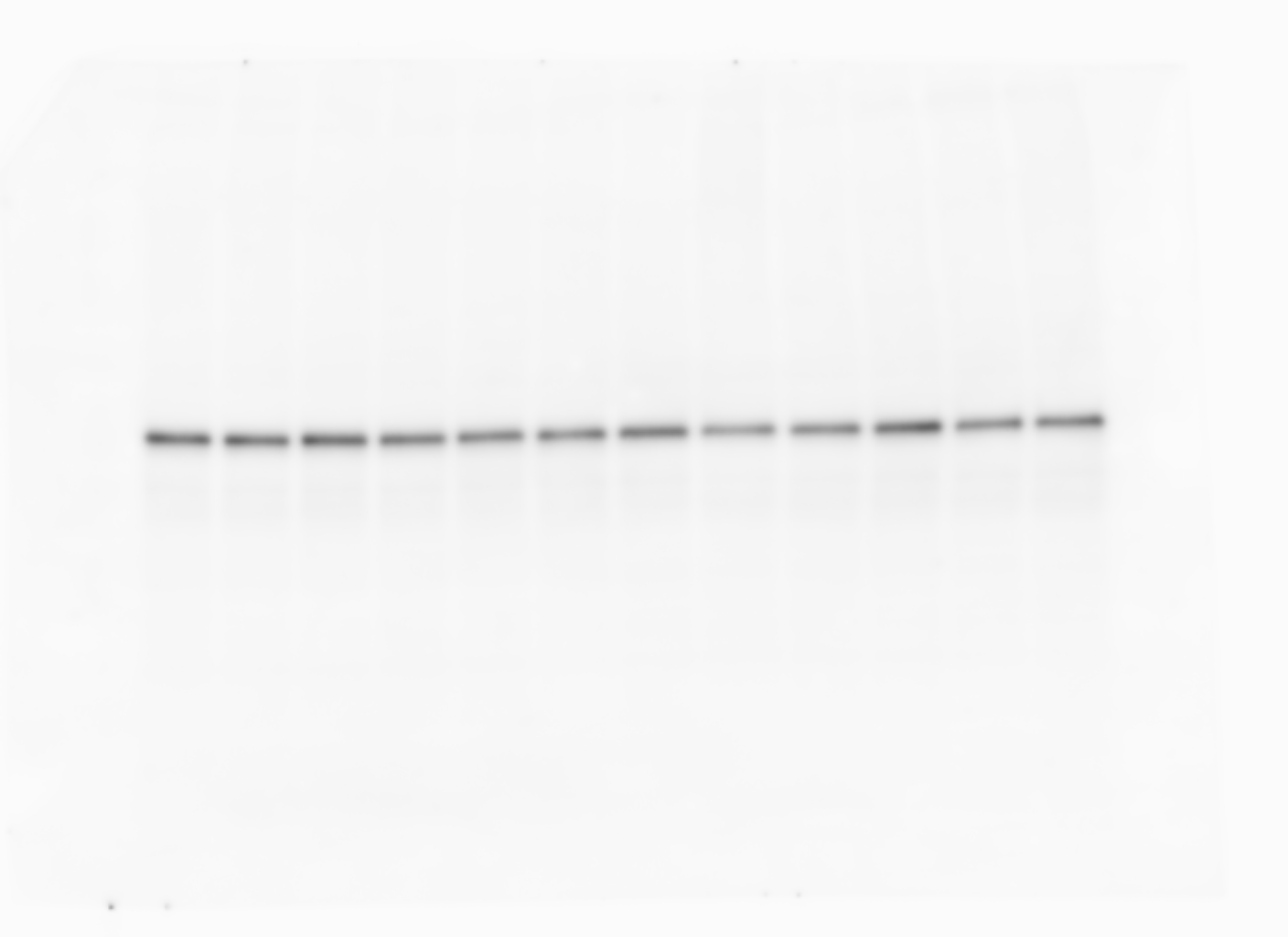

Supplement: Figure 6—source data 1. [file elife-88732-fig6-data1.zip › p-AKT Thr308/DR Akt Blot3 WPP 2018.02.01_12.07.20_Ch/DR Akt Blot3 WPP 2018.02.01_12.07.20_Ch.tif]

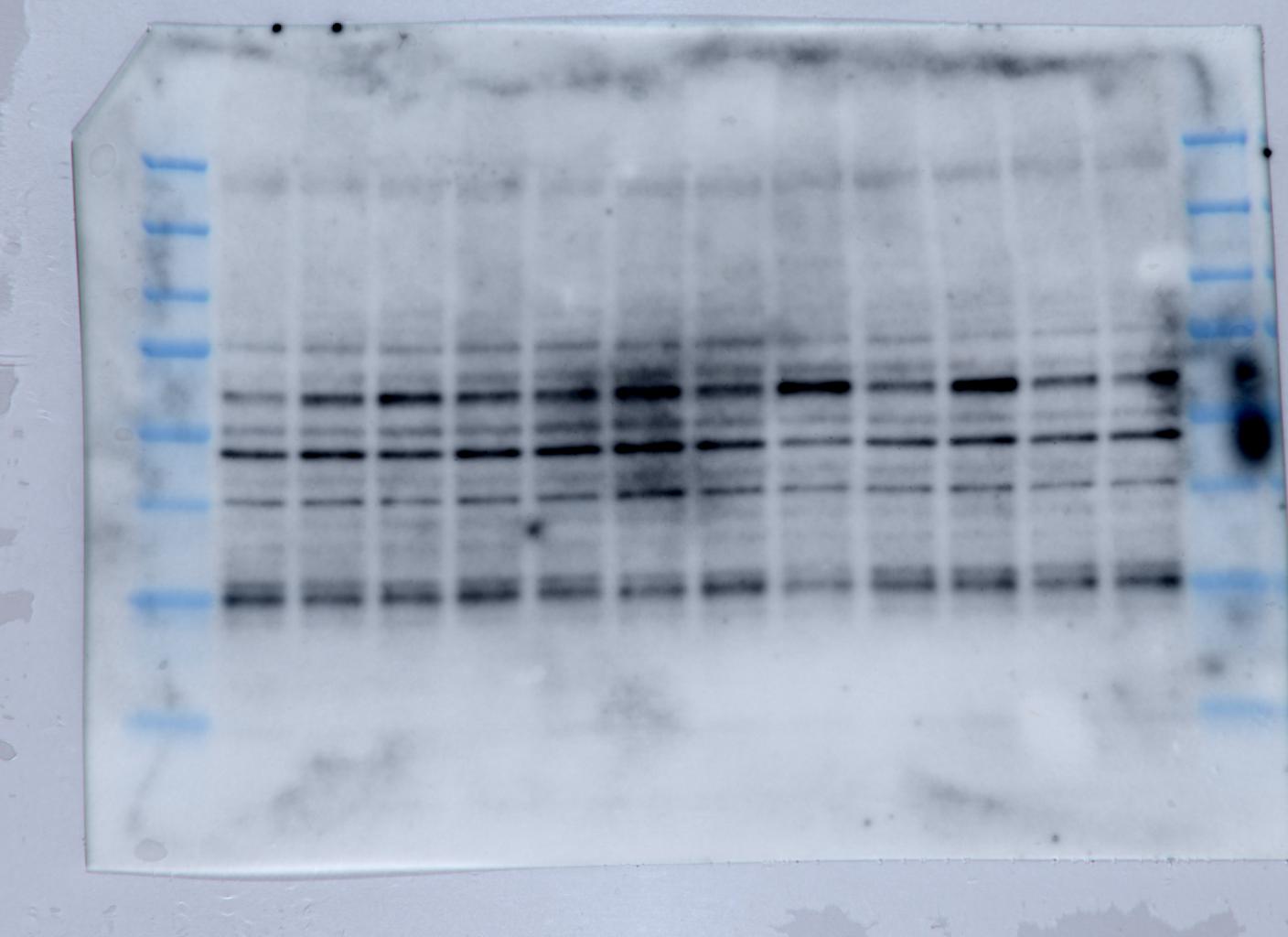

Supplement: Figure 6—source data 1. [file elife-88732-fig6-data1.zip › p-AKT Thr308/DR pAKT T308 WPP 2018.01.31_13.02.32_Ch/DR pAKT T308 WPP 2018.01.31_13.02.32_Ch+Marker.jpg]

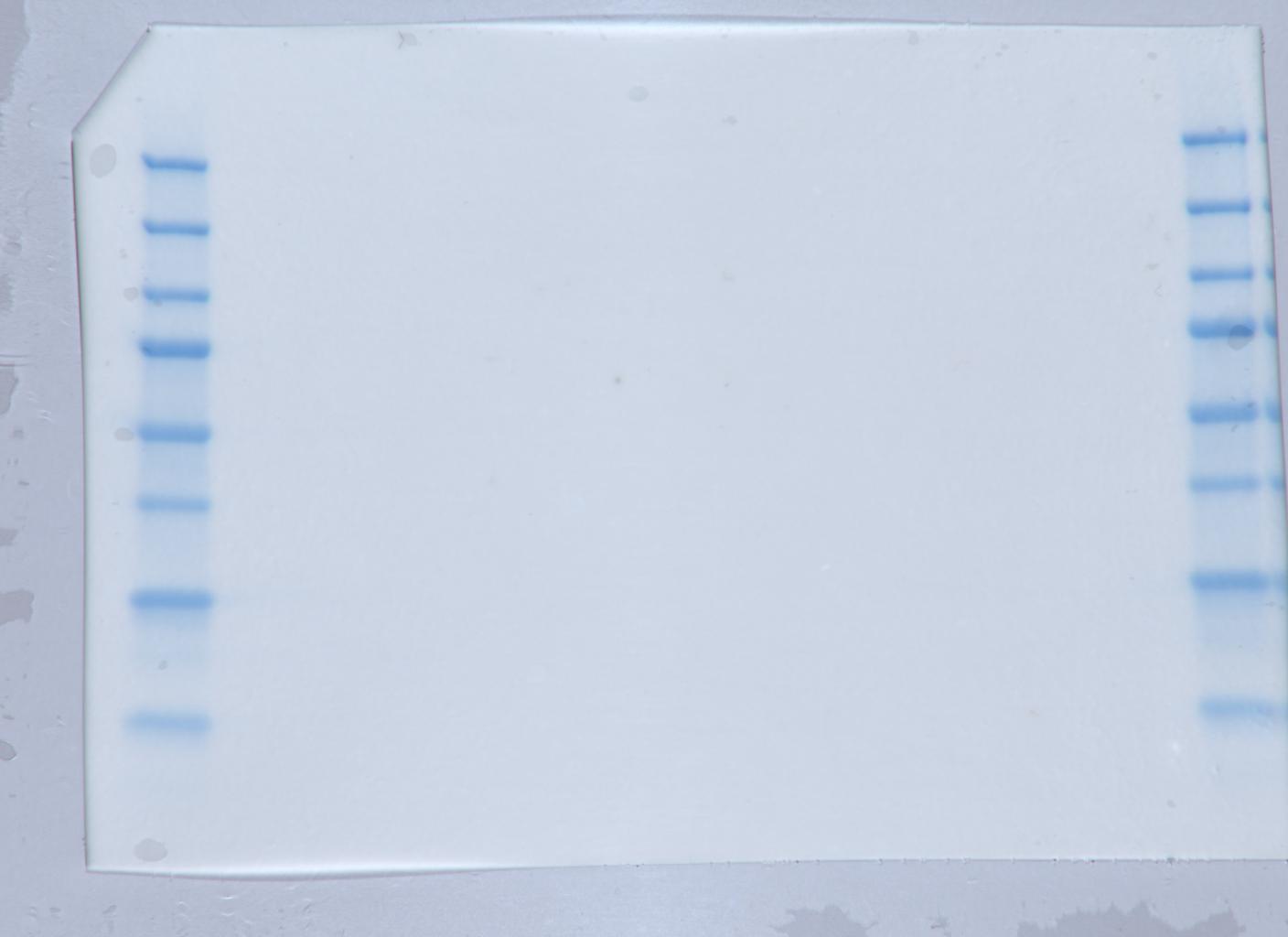

Supplement: Figure 6—source data 1. [file elife-88732-fig6-data1.zip › p-AKT Thr308/DR pAKT T308 WPP 2018.01.31_13.02.32_Ch/DR pAKT T308 WPP 2018.01.31_13.02.32_Ch-Marker.jpg]

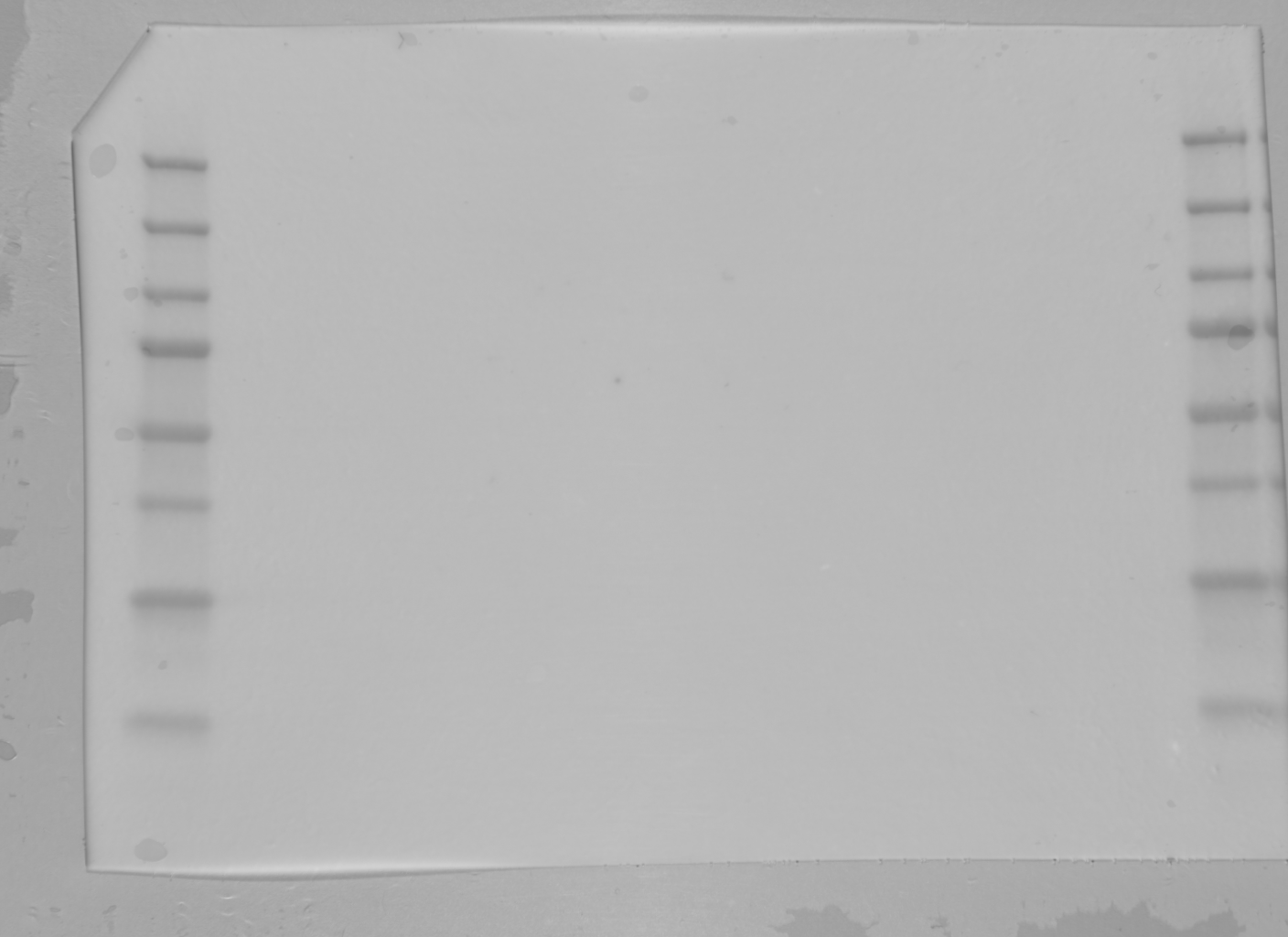

Supplement: Figure 6—source data 1. [file elife-88732-fig6-data1.zip › p-AKT Thr308/DR pAKT T308 WPP 2018.01.31_13.02.32_Ch/DR pAKT T308 WPP 2018.01.31_13.02.32_Ch-Marker.tif]

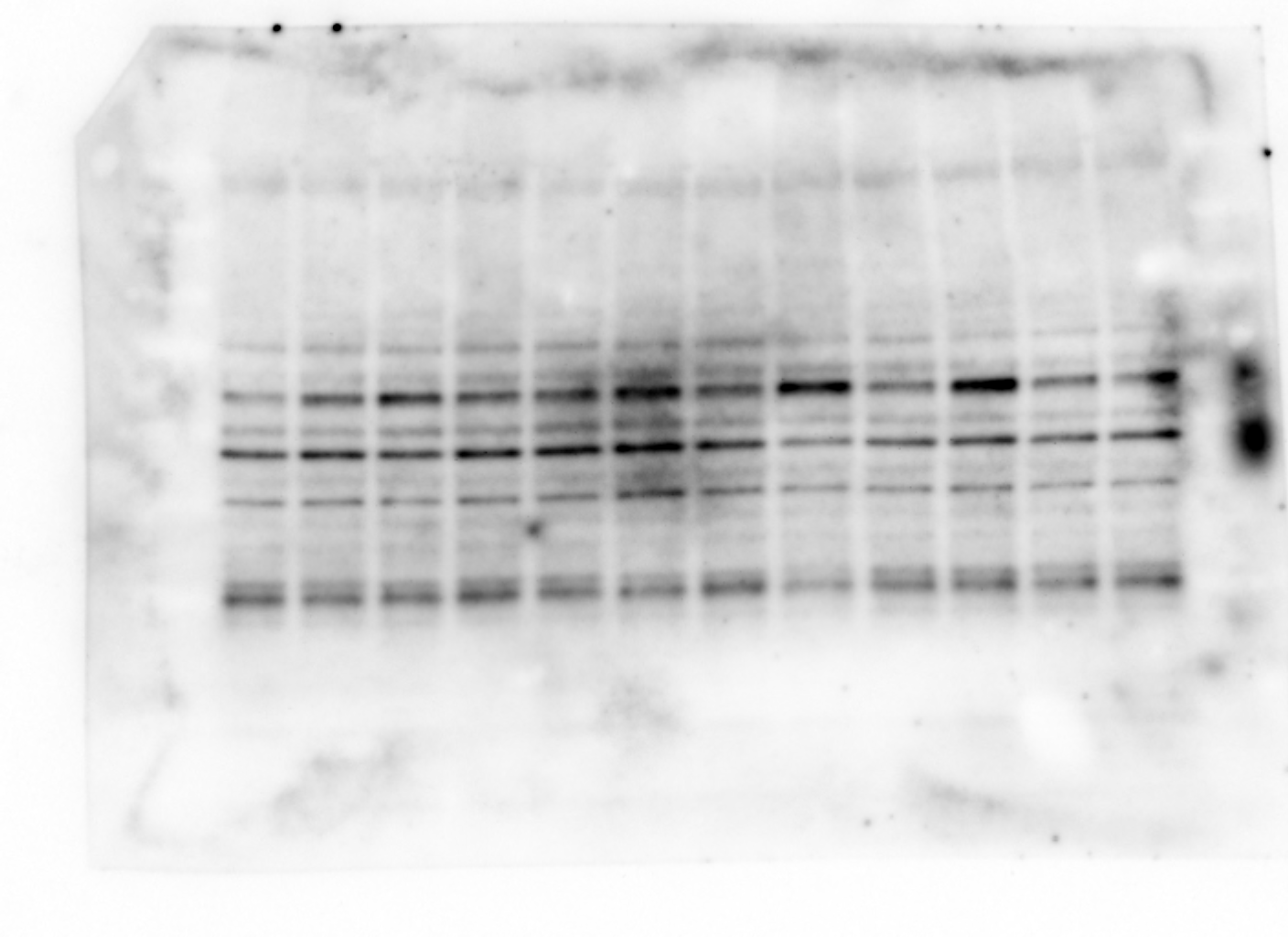

Supplement: Figure 6—source data 1. [file elife-88732-fig6-data1.zip › p-AKT Thr308/DR pAKT T308 WPP 2018.01.31_13.02.32_Ch/DR pAKT T308 WPP 2018.01.31_13.02.32_Ch.tif]

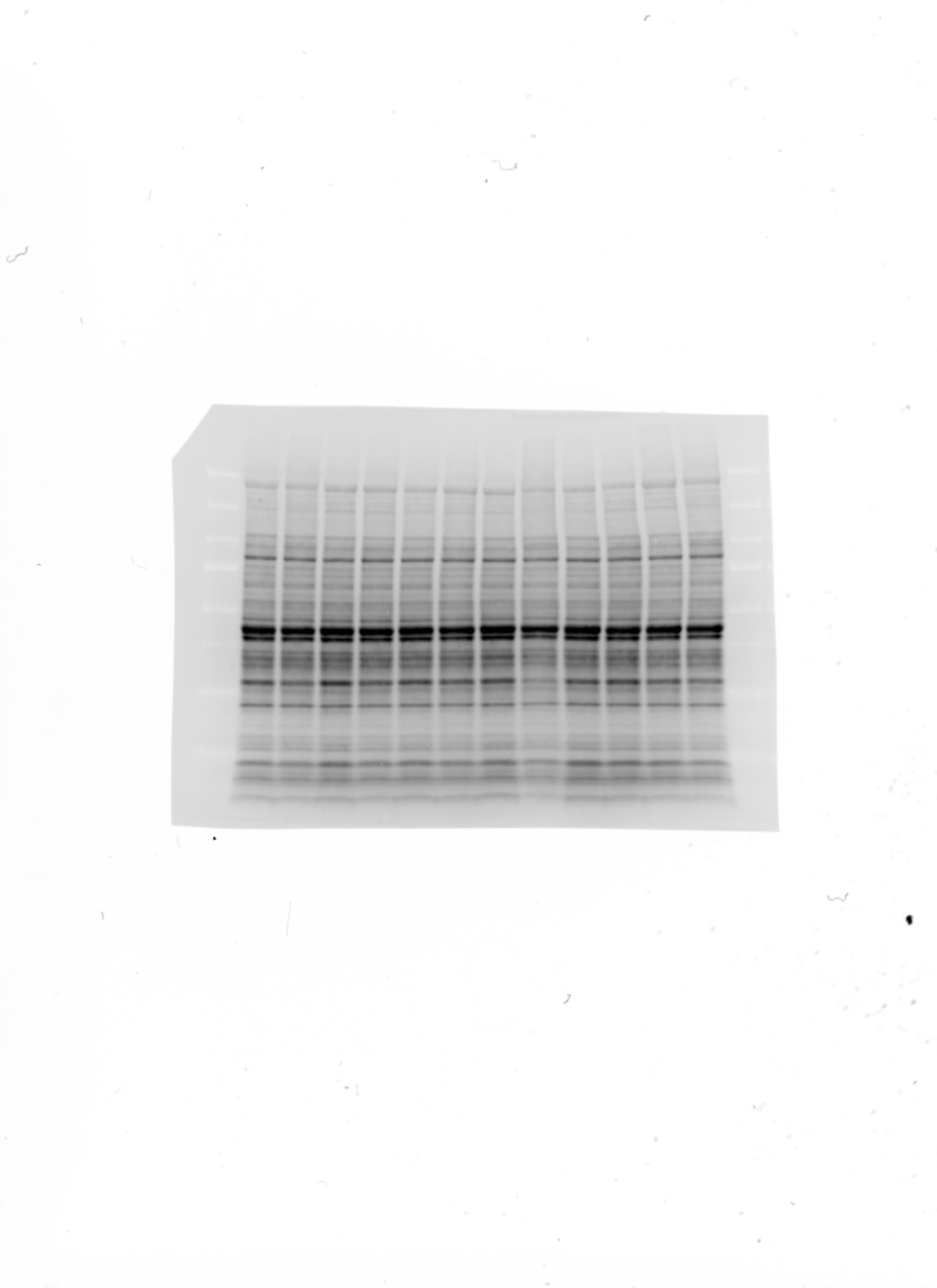

Supplement: Figure 6—source data 1. [file elife-88732-fig6-data1.zip › p-AKT Thr308/DR TotProt LV Blot3 2018.01.29_13.19.08_Fl-UV/DR TotProt LV Blot3 2018.01.29_13.19.08_Fl-UV.jpg]

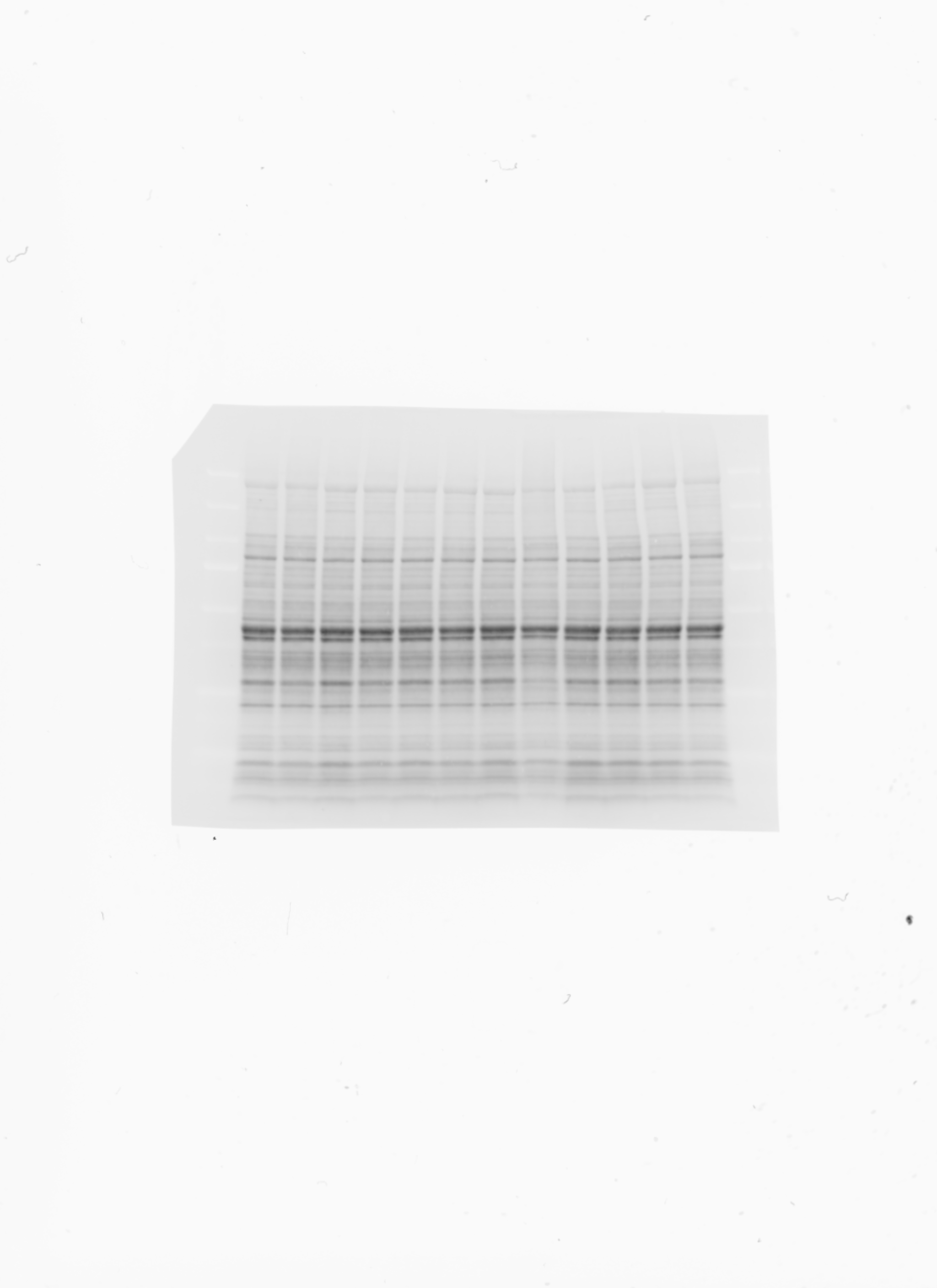

Supplement: Figure 6—source data 1. [file elife-88732-fig6-data1.zip › p-AKT Thr308/DR TotProt LV Blot3 2018.01.29_13.19.08_Fl-UV/DR TotProt LV Blot3 2018.01.29_13.19.08_Fl-UV.tif]

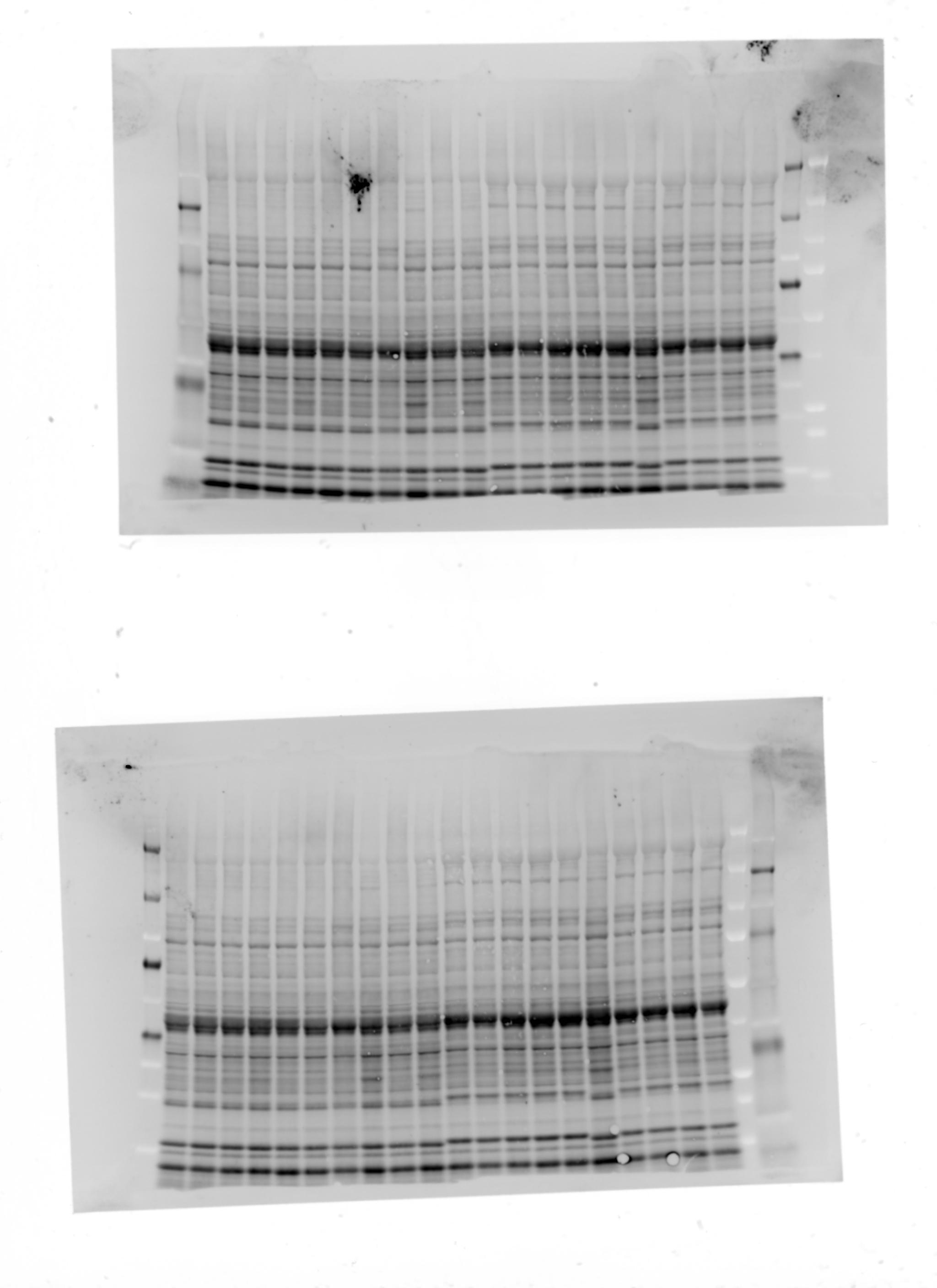

Supplement: Figure 6—source data 2. [file elife-88732-fig6-data2.zip › MGP_TotProt1-2 2023.01.11_16.05.11_Fl-UV.jpg]

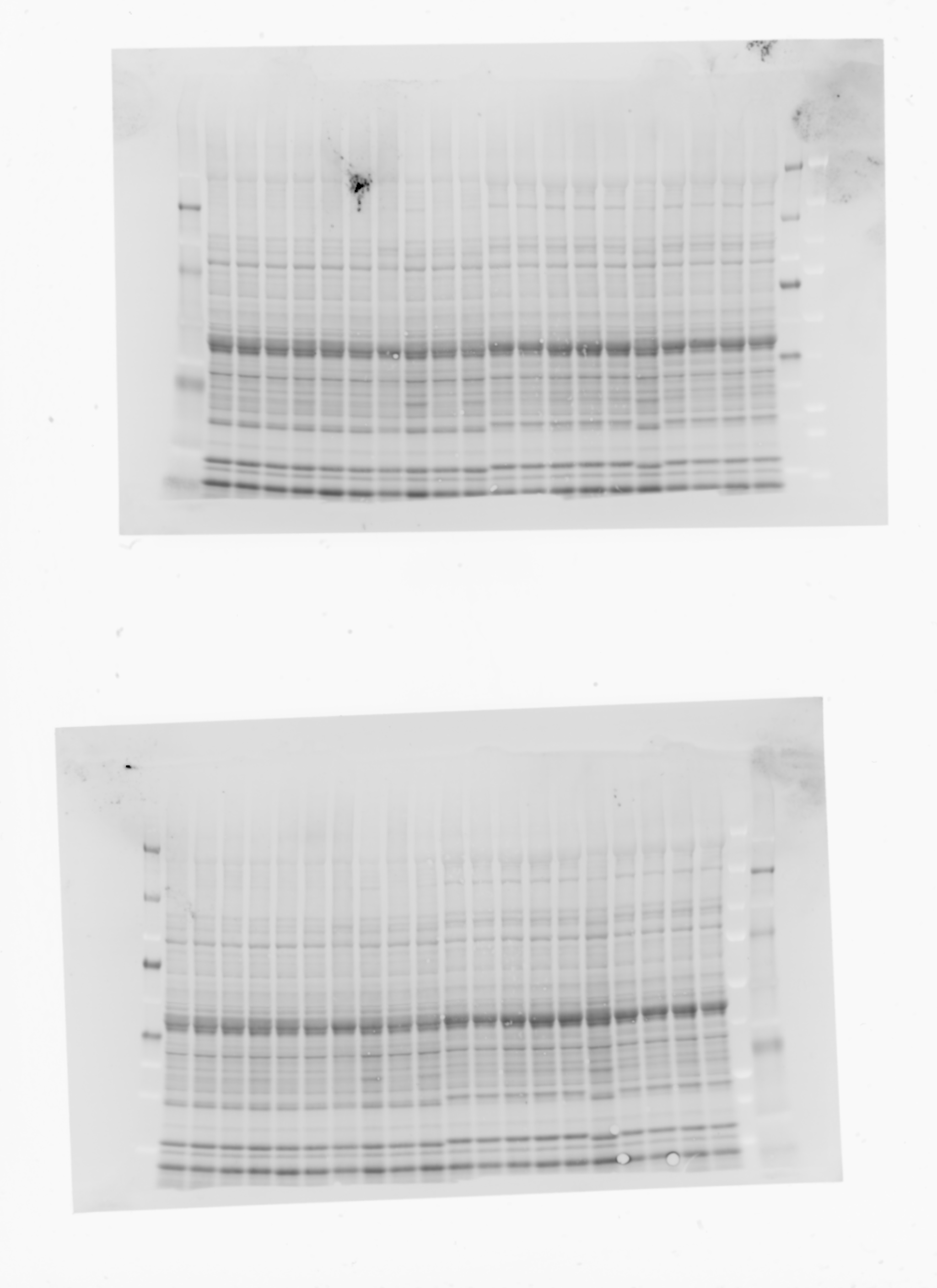

Supplement: Figure 6—source data 2. [file elife-88732-fig6-data2.zip › MGP_TotProt1-2 2023.01.11_16.05.11_Fl-UV.tif]

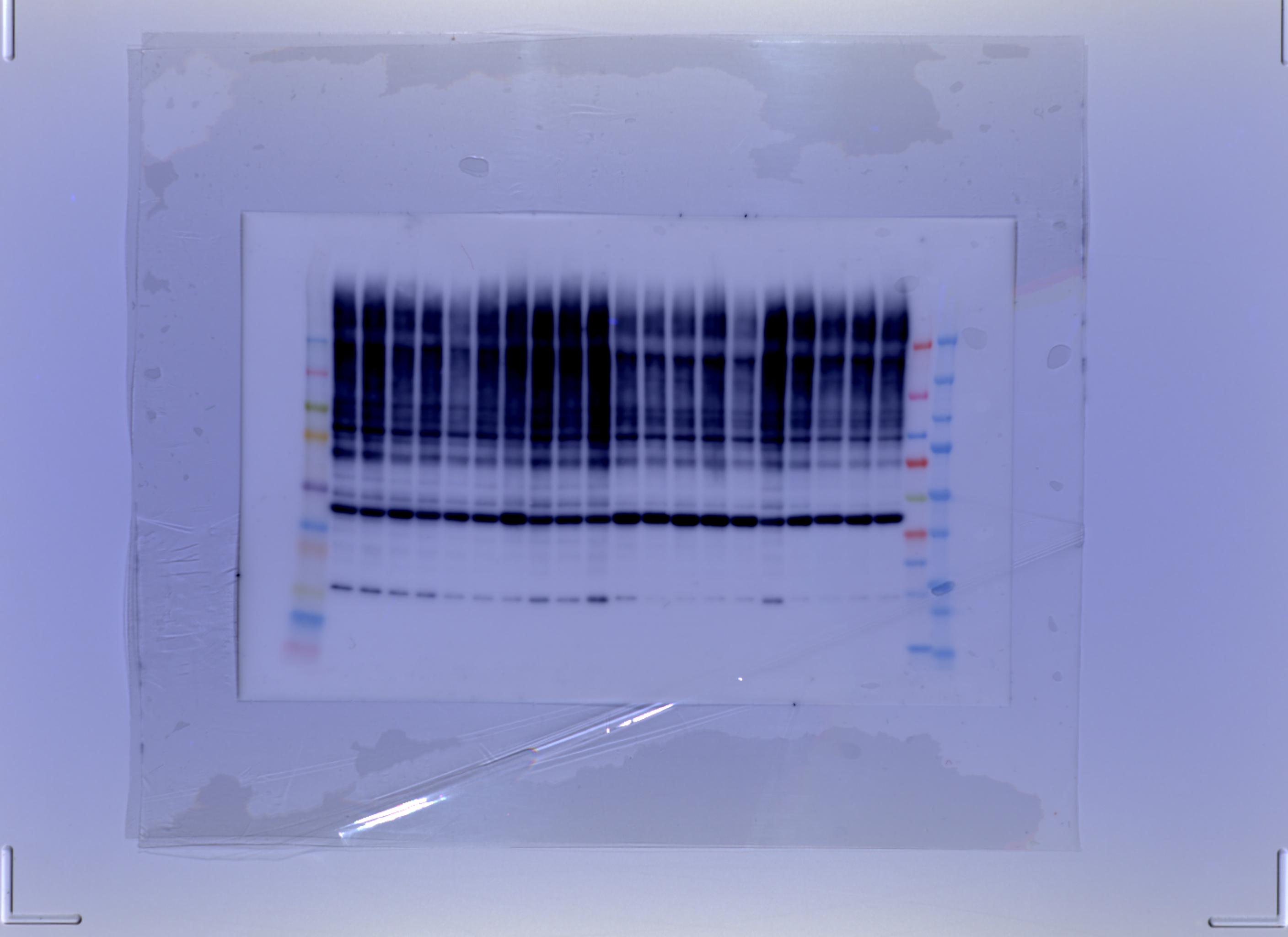

Supplement: Figure 6—source data 2. [file elife-88732-fig6-data2.zip › MGP_UbfrK48 Snp 10+Y 2023.01.13_13.38.05_Ch+Marker.jpg]

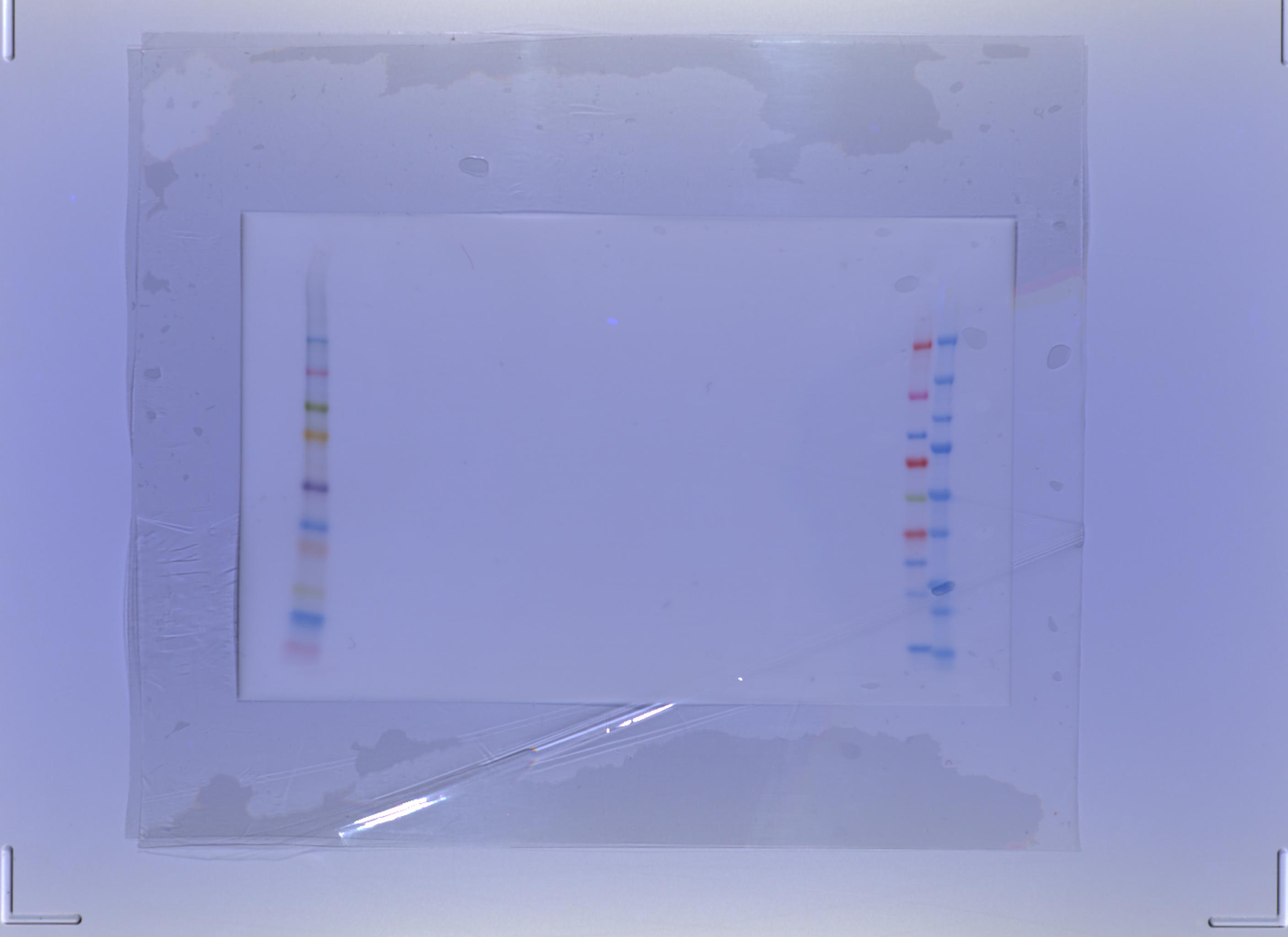

Supplement: Figure 6—source data 2. [file elife-88732-fig6-data2.zip › MGP_UbfrK48 Snp 10+Y 2023.01.13_13.38.05_Ch-Marker.jpg]

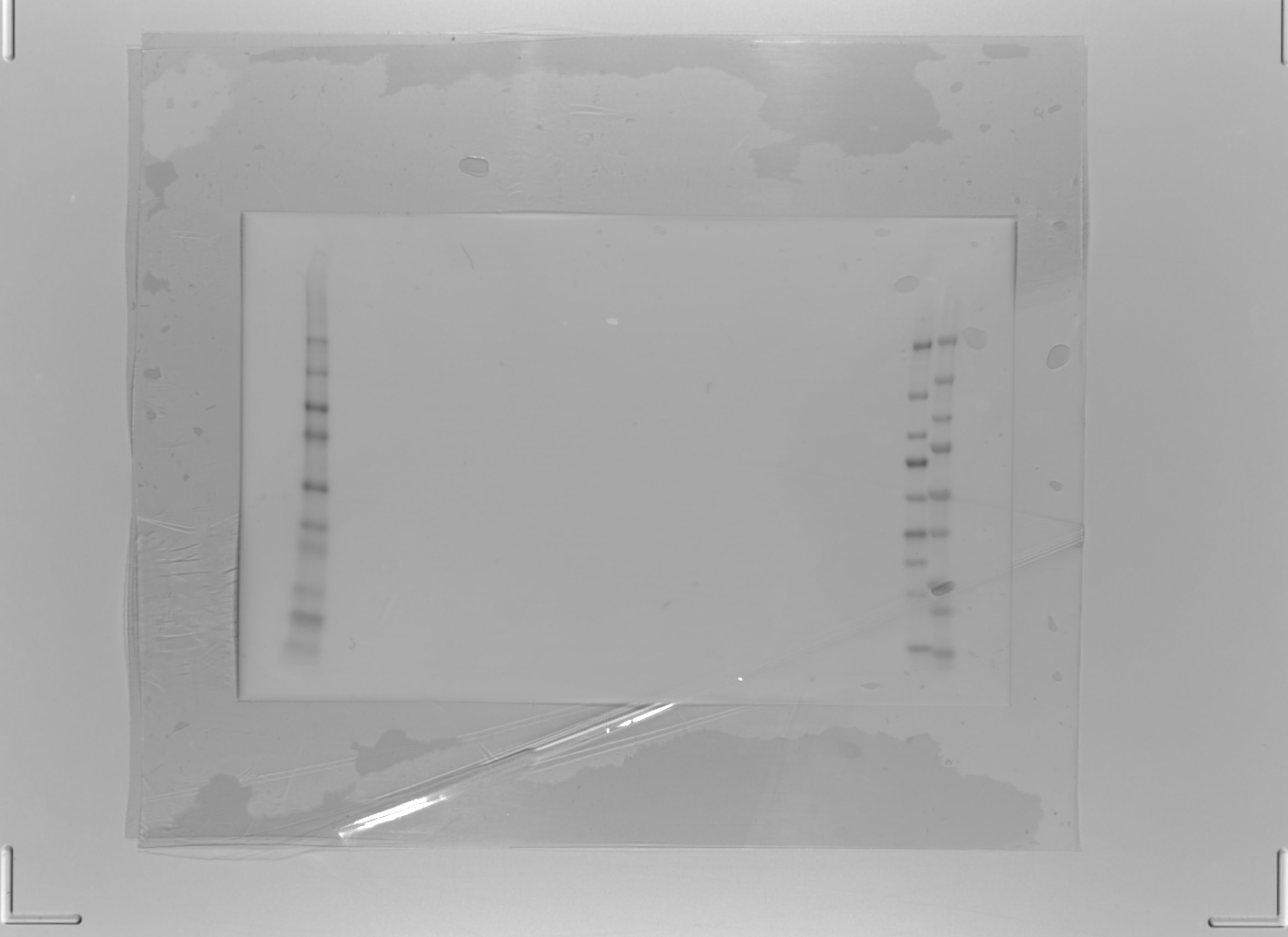

Supplement: Figure 6—source data 2. [file elife-88732-fig6-data2.zip › MGP_UbfrK48 Snp 10+Y 2023.01.13_13.38.05_Ch-Marker.tif]

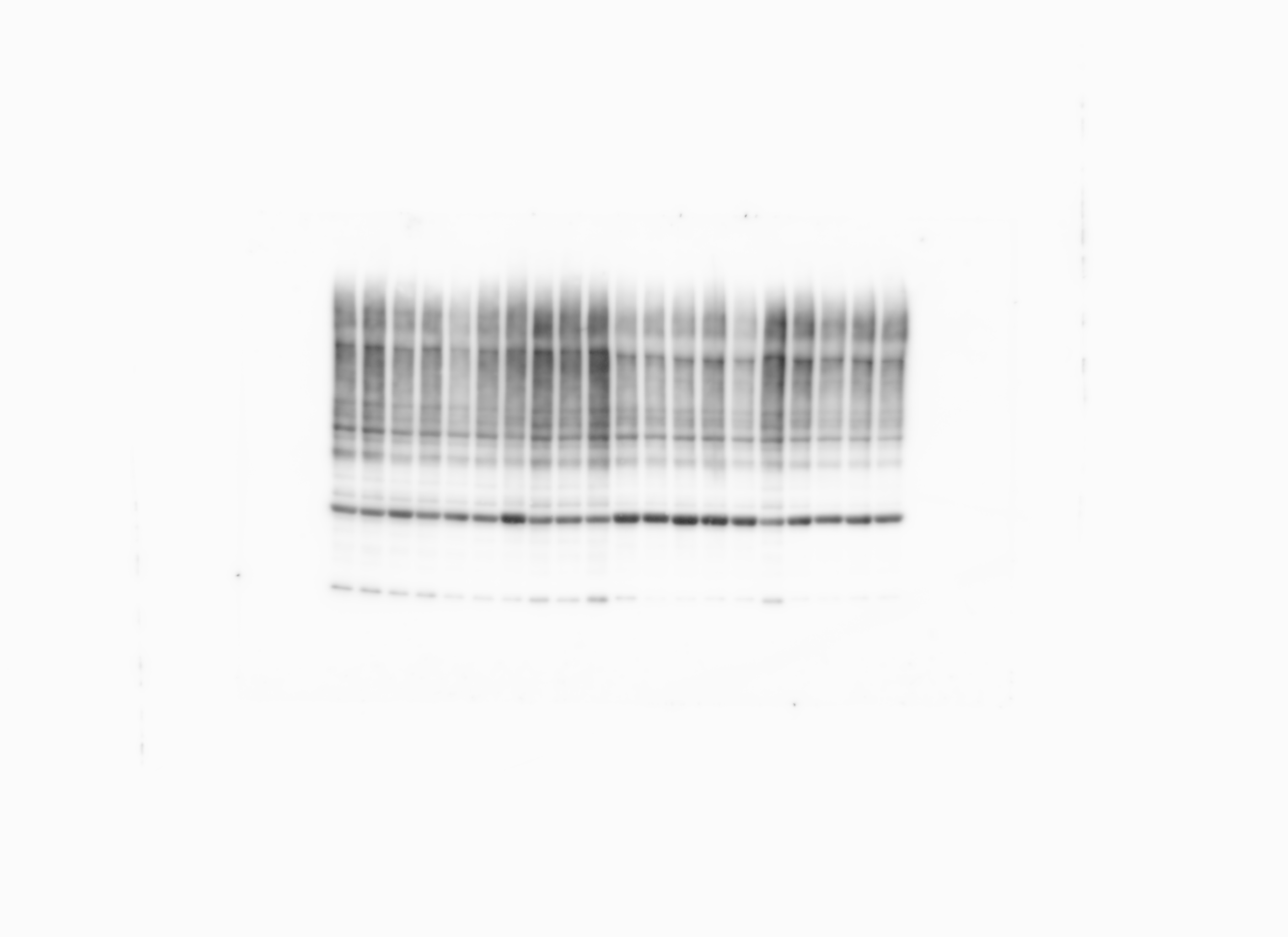

Supplement: Figure 6—source data 2. [file elife-88732-fig6-data2.zip › MGP_UbfrK48 Snp 10+Y 2023.01.13_13.38.05_Ch.tif]
