# Supplementary material for: Reprogramming of cardiac phosphoproteome, proteome, and transcriptome confers resilience to chronic adenylyl cyclase-driven stress
Source: eLife. 2024 Jan 22;12:RP88732. doi: 10.7554/eLife.88732 (PMC10945681; doi:10.7554/eLife.88732)
Supplement: Figure 6—source data 2. [file elife-88732-fig6-data2.zip › Ubiquitination data_eLIFE REQUEST.pptx]

## Slide 1
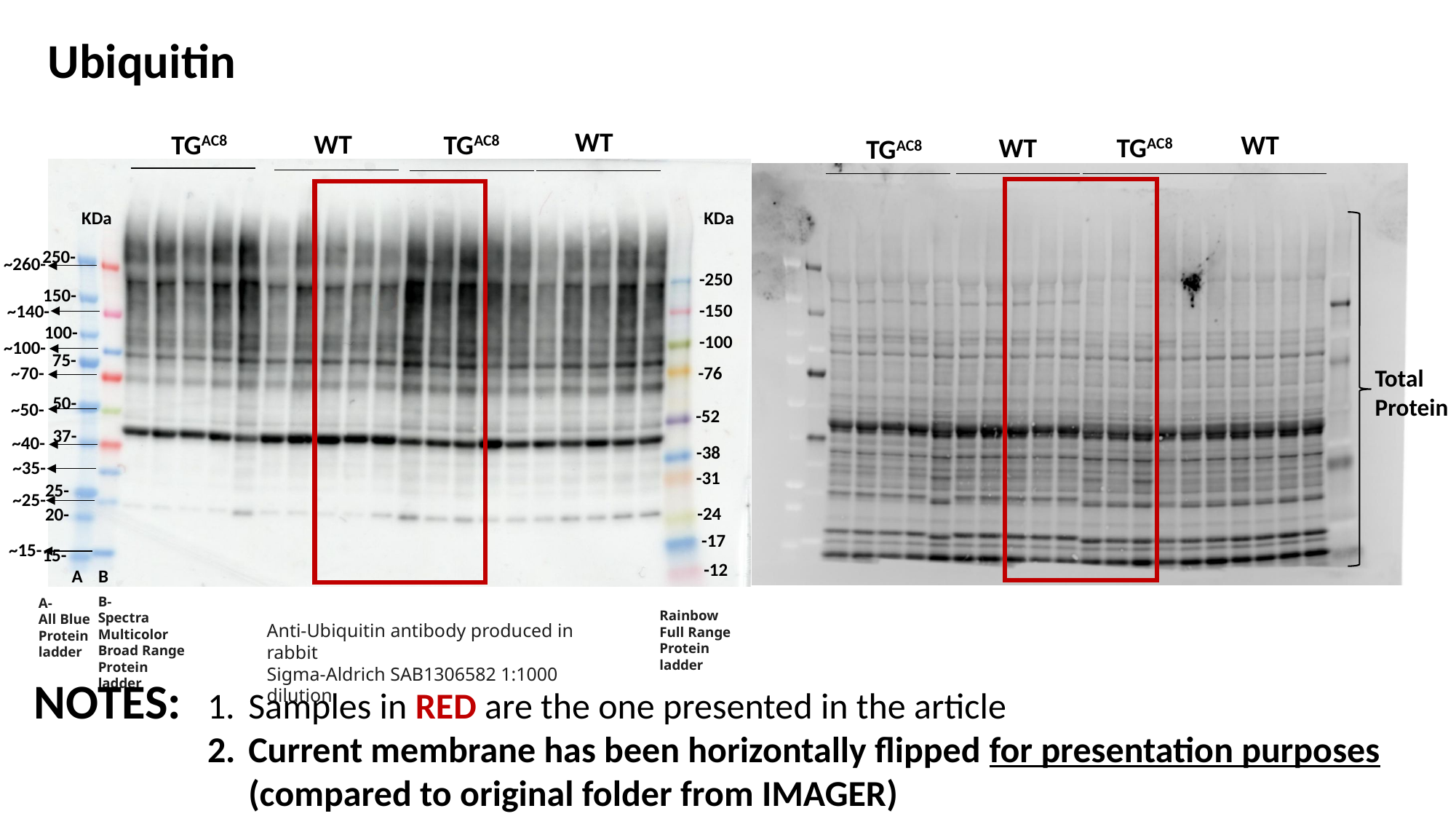

Ubiquitin
WT
WT
TGAC8
TGAC8
KDa
KDa
250-
~260-
-250
150-
-150
~140-
100-
-100
~100-
75-
~70-
-76
50-
~50-
-52
37-
~40-
-38
~35-
-31
25-
~25-
-24
20-
-17
~15-
15-
-12
A
B
B-
Spectra Multicolor
Broad Range
Protein
ladder
A-
All Blue
Protein
ladder
Rainbow Full Range
Protein ladder
Anti-Ubiquitin antibody produced in rabbit
Sigma-Aldrich SAB1306582 1:1000 dilution
WT
TGAC8
WT
TGAC8
Total
Protein
NOTES:
Samples in RED are the one presented in the article
Current membrane has been horizontally flipped for presentation purposes (compared to original folder from IMAGER)
